# Safety and Efficacy of Efgartigimod for Generalized Myasthenia Gravis: A Systematic Review and Meta‐Analysis

**DOI:** 10.1002/brb3.71875

**Published:** 2026-11-11

**Authors:** Ghazi Uddin Ahmed, FNU Ramsha, Sejal Kinger, Jawad Ghias Shaikh, FNU Prejna, FNU Ritik, Iqra Shabir, Faisal Suhail, Muhammad Abdul Basit, Muhammad Moiz, Amjad Ali, Abdul Manan, Abedin Samadi, Ahmed Asad Raza

**Affiliations:** ^1^ Department of Medicine Jinnah Sindh Medical University Karachi Pakistan; ^2^ Chandka Medical College Larkana Pakistan; ^3^ Department of Medicine Ghulam Muhammad Mahar Medical College Sukkur Pakistan; ^4^ Department of Medicine Dow University of Health Sciences Karachi Pakistan; ^5^ Chandka Medical College, SMBBMU Larkana Pakistan; ^6^ Department of Medicine Kabul University of Medical Sciences Abu Ali Sina Kabul Afghanistan

**Keywords:** efgartigimod, FcRn antagonist, generalized myasthenia gravis

## Abstract

**Background:**

Severe muscle weakness is a hallmark of generalized myasthenia gravis (gMG), a chronic autoimmune disease that severely reduces quality of life. Traditional immunosuppressive treatments can have significant adverse effects and only partially relieve symptoms. Clinical trials have demonstrated the potential of efgartigimod, a neonatal Fc receptor (FcRn) antagonist that lowers harmful IgG autoantibodies. This systematic review and meta‐analysis assessed its efficacy and safety, analyzing randomized controlled evidence separately from uncontrolled real‐world evidence.

**Methods:**

Up until August 2025, we conducted a thorough search of PubMed, Scopus, and Web of Science, supplemented by the Cochrane Central Register of Controlled Trials (CENTRAL) and ClinicalTrials.gov, for observational studies and randomized controlled trials (RCTs) evaluating efgartigimod in gMG. Improvement in myasthenia gravis activities of daily living (MG‐ADL), responder rates, adverse events, and quantitative myasthenia gravis (QMG) scores were the main outcomes. Placebo‐controlled RCTs were pooled in two‐arm meta‐analyses (primary evidence) and uncontrolled cohort studies and case series in single‐arm meta‐analyses (supportive evidence), using random‐effects models in Review Manager 5.4 and R (v4.4.2). Sensitivity, leave‐one‐out influence, and subgroup analyses were performed, 95% prediction intervals were calculated for single‐arm outcomes, and the certainty of evidence was rated using the Grading of Recommendations, Assessment, Development and Evaluation (GRADE) approach. The review was retrospectively registered in PROSPERO (CRD420261518373).

**Results:**

We included 11 studies (two RCTs, six cohort studies, and three case series) comprising 480 participants (385 efgartigimod‐treated and 95 placebo‐treated). In the RCTs, efgartigimod improved MG‐ADL (mean differences [MD] = −2.62, 95% CI: −3.99 to −1.25, *p* = 0.0002) and QMG scores (MD = −4.59, 95% CI: −6.24 to −2.94, *p* < 0.00001) at peak effect, but the differences were not significant at the end of follow‐up. MG‐ADL responders were more frequent with efgartigimod than with placebo (risk ratios [RR] = 1.90, 95% CI: 1.40–2.56, *p* < 0.0001), including among acetylcholine receptor (AChR)‐Ab+ patients (RR = 2.37, 95% CI: 1.61–3.47, *p* < 0.0001). The incidence of adverse events did not differ from placebo (RR = 0.93, 95% CI: 0.81–1.07, *p* = 0.29). Certainty of evidence was moderate for peak‐effect, responder, and safety outcomes. In uncontrolled real‐world studies, responses were observed in AChR‐Ab+ and muscle‐specific tyrosine kinase (MuSK)‐Ab+ patients (MuSK‐Ab+ pooled proportion = 0.781, 95% CI: 0.499–0.927; 18 patients) and corticosteroid doses decreased, but this evidence was of very low certainty. Single‐arm estimates showed substantial to considerable heterogeneity (*I* ^2^ up to 98.8%) and wide 95% prediction intervals, although every contributing study showed improvement in MG‐ADL and a reduction in corticosteroid dose. Randomized estimates were unchanged when restricted to the phase 3 ADAPT trial, which contributed 71%–92% of the statistical weight.

**Conclusion:**

In randomized trials, efgartigimod produces clinically meaningful short‐term improvement in gMG with an adverse event incidence comparable to placebo. Because the benefit was not maintained at the end of follow‐up, cyclical, individualized retreatment is likely required. Real‐world data suggest benefit across serological subtypes and a corticosteroid‐sparing effect; however, these uncontrolled estimates were heterogeneous and of very low certainty, and larger, longer term controlled studies, ideally conducted independently of the manufacturer, are needed to confirm these findings.

## Graphical abstract

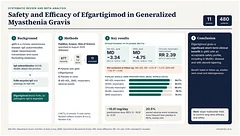

## 1 Introduction

Generalized myasthenia gravis (gMG) is a chronic autoimmune illness that causes severe muscle weakness in the eyes, bulbar regions, limbs, and respiratory system. This disorder can result in considerable disability and a decreased quality of life as affected patients experience gradual and unexpected disease course (Gilhus et al. 2019). About 85% of patients with gMG have IgG autoantibodies, which are mostly directed against the acetylcholine receptor (AChR) while other detected antibodies in gMG affected patients against low‐density lipoprotein receptor‐related protein 4 (LRP4) or muscle‐specific tyrosine kinase (MuSK) are less frequently seen (Zisimopoulou et al. 2014; Gilhus and Verschuuren 2015). By decreasing AChR availability and inducing complement‐mediated postsynaptic membrane damage, AChR antibodies mostly IgG1 and IgG3 subtypes hinder neuromuscular transmission.

MuSK antibodies, on the other hand, are primarily IgG4 and interfere with agrin‐LRP4‐MuSK interactions, which is essential for synaptic integrity (Punga et al. 2022). Therapies like corticosteroids and nonsteroidal immunosuppressants (NSISTs) suppress immune function generally Instead of specifically targeting pathogenic IgG, hence, requiring weighing benefits against risks that may occur. Although some patients have symptomatic relief from these treatments, their effectiveness is frequently insufficient. Moreover, patients who use the traditional treatment options for long are prone to metabolic abnormalities, cardiovascular risks, and osteoporosis (Skeie et al. 2010). Although complement inhibitors, such as eculizumab, provide a more focused treatment for AChR‐positive gMG (Howard 2018; Howard et al. 2017).

IgG's half‐life is roughly four times longer than that of other immunoglobulins that are not recycled by the neonatal Fc receptor (FcRn), such as IgM or IgA (Kuo et al. 2010). An IgG antibody's Fc region interacts to two FcRn receptors in the endosome under acidic circumstances after cellular absorption (Jensen et al. 2017). After being saved from lysosomal breakdown, IgG coupled to FcRn is released from the cell at physiological pH (Ghetie et al. 1996; Junghans and Anderson 1996; Roopenian and Akilesh 2007; Ward 2003). Thus, in IgG‐mediated disorders like gMG, FcRn maintains the availability of IgG autoantibodies. The usefulness of immunoadsorption and plasma exchange in eliminating autoantibodies in gMG has been shown, but their application is restricted due to their availability and need for specializing facilities (Liu et al. 2010). Targeting the primary pathogenic driver in gMG by blocking FcRn is a sensible treatment strategy.

Neonatal FcRn antagonists decrease IgG recycling, which lowers pathogenic antibodies and improves gMG clinically. One of the best neonatal FcRn antagonists is efgartigimod. Compared to 30% of patients getting a placebo, 68% of patients receiving efgartigimod therapy demonstrated improvement in myasthenia gravis activities of daily living (MG‐ADL) in a phase 3 randomized controlled trial (RCT) (ADAPT trial) (Howard et al. 2021). Efgartigimod was initially authorized in the United States in December 2021 for the treatment of gMG in adults who are AChR antibody‐positive, based on the findings of clinical trials (Howard et al. 2019, 2021).

At least four previous systematic reviews have pooled data on efgartigimod in myasthenia gravis. Three included only placebo‐controlled RCTs: a pairwise and network meta‐analysis of innovative therapies (Saccà et al. 2023), a meta‐analysis of FcRn inhibitors (Li et al. 2024), and a network meta‐analysis of targeted biological therapies that examined quantitative myasthenia gravis (QMG) change at several time points, including after the last dose (Zhong et al. 2024). Because only two placebo‐controlled trials of efgartigimod have been published, the efgartigimod estimates in these reviews were derived from one or both of the same trials, which enrolled predominantly AChR‐Ab+ patients treated with a fixed cycle. The fourth, a recent meta‐analysis focused on refractory disease (Cheng et al. 2025), combined randomized and observational studies, pooling responder and adverse event proportions. We do not regard the short‐term efficacy of efgartigimod over placebo as an open question. However, none of these reviews pooled outcomes in MuSK‐Ab+ patients or changes in corticosteroid dose, compared both MG‐ADL and QMG at peak effect and at the end of follow‐up, analyzed randomized and uncontrolled evidence as separate tiers for every outcome, or reported a Grading of Recommendations, Assessment, Development and Evaluation (GRADE) assessment of the certainty of evidence. Several questions that matter for clinical decision‐making thus remain incompletely answered: whether the benefit observed at peak response persists to the end of follow‐up, whether patients with MuSK‐Ab+ or seronegative disease respond, whether efgartigimod permits corticosteroid tapering in routine practice, and how much confidence can be placed in each of these estimates. We therefore conducted a systematic review and meta‐analysis that (i) analyzes placebo‐controlled RCTs and uncontrolled real‐world studies as distinct tiers of evidence, drawing comparative inferences only from randomized data; (ii) evaluates outcomes both at peak effect and at the end of follow‐up; (iii) examines serotype‐specific responses and corticosteroid‐sparing effects in real‐world cohorts; and (iv) assesses the robustness of pooled estimates through sensitivity and influence analyses and rates the certainty of evidence using the GRADE approach.

## 2 Methods

### 2.1 Protocol and Registration

This systematic review adhered to the Preferred Reporting Items for Systematic Reviews and Meta‐Analyses (PRISMA) statement standards (Moher et al. 2010). The review protocol was registered in the International Prospective Register of Systematic Reviews (PROSPERO; registration number CRD420261518373). Registration was completed retrospectively, after the literature search had been performed, and we acknowledge this as a limitation (Section 4.3). To reduce the risk of selective reporting, the eligibility criteria, outcomes, and analytic methods are reported in full, results are presented for every outcome analyzed, and the complete search strategy is provided in Table S1. Because registration followed the search, none of the analyses can be regarded as prospectively pre‐specified. We therefore label the sensitivity, influence, and subgroup analyses, the prediction intervals (PI), and the supplementary searches as post hoc; report results for every outcome and analysis performed; and provide the study‐level data extracted for each meta‐analysis in the Supporting Information so that all analyses can be reproduced. The study selection process is reported using the PRISMA 2020 flow diagram (Page et al. 2021).

### 2.2 Literature Search and Study Selection

Without regard to language limitations, we carried out a thorough search of the Web of Science, PubMed, and Scopus databases for articles published up until August 8, 2025. We applied the following terms (“Myasthenia Gravis”) AND (efgartigimod OR “ARGX‐113”) and manually searched for other relevant studies. We tabulated search strategies in Table S1. To determine which papers satisfied the inclusion and exclusion criteria, two different authors independently examined the search results. They also assessed each study's qualifying criteria. After the initial screening of abstracts and titles, we acquired the complete texts of potentially pertinent papers for a more thorough analysis. Any disputes that arose throughout the procedure were settled by consensus.

Because the original search did not include a dedicated register of controlled trials, we performed supplementary searches of the Cochrane Central Register of Controlled Trials (CENTRAL; Cochrane Library) and the ClinicalTrials.gov registry during peer review, on October 12, 2026. These searches combined MeSH and free‐text terms for myasthenia gravis and for efgartigimod (efgartigimod, ARGX‐113, and Vyvgart) and were limited to records published or first posted up to August 8, 2025, the end date of the original search, so that the evidence base remained consistent (Table S1). Retrieved records were checked against those identified in the original search and screened independently by two reviewers using the same eligibility criteria. Registry entries were used to identify completed or ongoing placebo‐controlled trials and their corresponding publications.

### 2.3 Eligibility Criteria

We included studies that met the following PICO criteria:

1. Population: Patient with gMG.
2. Intervention: Efgartigimod.
3. Comparator: Placebo for RCTs. Observational studies without a control group were also eligible and contributed to single‐arm analyses only.
4. At least one of the following outcomes: MG‐ADL improvement, QMG improvement, clinically meaningful improvement achievement, MG‐ADL responders, AChR+ responders, adverse events, QMG responders, MuSK+ responders, clinical improvement, and prednisolone dosage reduction
5. Study design: RCTs and observational studies (prospective or retrospective cohort studies and case series).

We discarded studies that did not meet our PICO criteria and were not conducted in the target population. We excluded studies reporting incomplete data.

### 2.4 Data Extraction

Using a standardized data extraction form, two researchers independently extracted the data in accordance with the Cochrane Handbook. A third researcher was consulted to see if there were any discrepancies in the data extraction.

The extracted data included the following:

- Study characteristics: Year of publication, country of origin, study design, aim of study, sample size, primary endpoints, duration of follow‐up, and methodology.
- Participant characteristics: Age distribution, gender demographics, inclusion and exclusion criteria, and baseline characteristics.
- Disease details: Disease duration; prior thymectomy baseline clinical assessment scores including MG‐ADL; QMG; myasthenia gravis composite (MGC); Myasthenia Gravis Quality of Life 15‐item revised (MG‐QoL15r); prednisone dosage used; and antibodies status including AChR‐Ab+, MuSK‐Ab+, and immunoglobulins G.
- Outcomes measured: Clinical assessment scores improvement/change from baseline and rate of responders including MG‐ADL, QMG, clinically meaningful improvement achievement, change in prednisone dose (mg/day), adverse events, clinically meaningful improvement achievement, and responder rate in positive antibodies.

### 2.5 Quality Assessment

To assess the methodological quality and risk of bias for all included studies, two reviewers independently conducted an evaluation using standardized critical appraisal tools appropriate for each study design. Any discrepancies between reviewers were resolved through discussion and consensus or, if necessary, consultation with a third reviewer was done. RCTs were assessed using the Cochrane risk of bias tool 2 (RoB 2) (Sterne et al. 2019). For non‐randomized cohort studies, we used the Newcastle‐Ottawa Scale (NOS) (Wells et al. 2000). For case series, we used the Joanna Briggs Institute (JBI) Critical Appraisal Checklist for Case Series (Munn et al. 2020)

### 2.6 Statistical Analysis

We analyzed all included outcomes using R statistical program version 4.4.2 (2024‐10‐31 ucrt) and the meta package. For single‐arm meta‐analyses of binary outcomes, we employed the generalized linear mixed model with logit transformation, which is better used especially in case of extreme values. We studied single‐arm continuous outcomes as mean change from baseline and restricted maximum‐likelihood (REML) estimators for tau^2^. In double‐arm analyses, we used Review Manager 5.4 to calculate risk ratios (RR) using the Mantel‐Haenszel method and mean differences (MD) using the inverse‐variance method, both under a random‐effects model. Two‐arm continuous outcomes were analyzed at the time of peak effect and at the end of study follow‐up, as reported by each trial. We used *I* ^2^ and tau^2^ to assess heterogeneity, and interpreted *I* ^2^ according to the Cochrane Handbook (30%–60%, moderate; 50%–90%, substantial; 75%–100%, considerable heterogeneity) (Higgins et al. 2024).

Because only two RCTs were available, between‐trial variance could not be estimated reliably. For two‐arm analyses, *I* ^2^ and tau^2^ are therefore reported for completeness but were not interpreted as evidence of consistency; instead, we report the weight contributed by each trial and compare random‐effects estimates with fixed‐effect estimates and with estimates from the phase 3 ADAPT trial alone (Section 2.8). For single‐arm outcomes, we calculated 95% PIs using the method of Higgins et al. (2009), based on a *t* distribution with k − 2 degrees of freedom, to estimate the range within which the outcome of a new, comparable study would be expected to lie (IntHout et al. 2016). Because *I* ^2^ describes the proportion of observed variance attributable to between‐study differences rather than the absolute size of those differences, and approaches 100% when within‐study estimates are precise, as is common in meta‐analyses of single‐arm proportions (Borenstein et al. 2017; Migliavaca et al. 2022), we interpreted heterogeneity mainly through tau, the range of study‐specific estimates, and the PI. When heterogeneity was considerable (*I* ^2^ > 75%), pooled single‐arm estimates were interpreted as the average of heterogeneous study results rather than as a single common effect.

### 2.7 Evidence Hierarchy

Studies were grouped into three tiers of evidence according to design: Tier 1, placebo‐controlled RCTs; Tier 2, prospective or retrospective cohort studies; and Tier 3, case series. Only Tier 1 studies contributed to two‐arm (comparative) meta‐analyses, which form the basis of our inferences on efficacy and safety relative to placebo. Tier 2 and Tier 3 studies, which lacked a placebo or untreated comparator, were pooled in single‐arm meta‐analyses of within‐group change or proportions. These estimates describe outcomes in treated patients in routine practice and were interpreted as supportive rather than causal evidence. Pane et al. (2024) included an eculizumab‐treated arm; because eculizumab was not an eligible comparator, only the efgartigimod arm of this study was analyzed.

### 2.8 Sensitivity, Influence, and Subgroup Analyses and Clinical Heterogeneity

For two‐arm outcomes, we compared random‐effects with fixed‐effect models and repeated each analysis using the phase 3 ADAPT trial alone, which, with two trials, is equivalent to a leave‐one‐out analysis. For single‐arm outcomes, we performed leave‐one‐out influence analyses in which the pooled estimate was recalculated after omitting each study in turn. We also performed subgroup analyses by study design (cohort studies vs. case series), testing between‐subgroup differences with a chi‐square test when each subgroup contained at least two studies, and a sensitivity analysis restricted to good‐quality cohort studies (NOS score ≥ 7). Because Horiuchi et al. (2024) enrolled only double‐seronegative patients, we additionally repeated the relevant analyses after excluding this study. Clinical heterogeneity was assessed qualitatively by comparing study populations (antibody status, disease severity, and refractoriness), dosing schedules (fixed or individualized cycles), timing of outcome assessment, and responder definitions across studies (Table 1; Table S2). Meta‐regression and formal assessment of publication bias (funnel plots and Egger's test) were not performed because fewer than 10 studies contributed to each outcome.

**TABLE 1:** Summary of included studies, grouped by level of evidence.

Study	Country	Design (evidence tier)	Participants analyzed, *n* (efgartigimod/comparator)	Antibody status	Efgartigimod regimen	Follow‐up	Outcomes contributed
Tier 1: Randomized controlled trials (two‐arm meta‐analysis)							
(Howard et al. 2019)	Multinational (8 countries)	Phase 2 double‐blind, placebo‐controlled RCT	24 (12/12 placebo)	AChR‐Ab+	10 mg/kg IV, four infusions over 3 weeks	11 weeks	MG‐ADL, QMG, MG‐ADL responders/CMI, AChR‐Ab+ responders, AEs
ADAPT (Howard et al. 2021)	North America, Europe, Japan	Phase 3 double‐blind, placebo‐controlled RCT	167 (84/83 placebo)	AChR‐Ab+ (*n* = 129) and AChR‐Ab‐ (*n* = 38)	10 mg/kg IV weekly × 4 per cycle; retreatment by clinical need	26 weeks	MG‐ADL^a^, QMG^a^, MG‐ADL responders/CMI, AChR‐Ab+ responders, AEs
Tier 2: Cohort studies (single‐arm meta‐analysis)							
(Frangiamore et al. 2024)	Italy	Prospective observational (expanded access)	19 (19/‐)	Mixed (AChR‐Ab+ 8; MuSK‐Ab+ 4)	10 mg/kg IV × 4; two fixed cycles, then flexible	14 months	MG‐ADL, QMG, MG‐ADL and QMG responders, AChR‐Ab+ and MuSK‐Ab+ responders, CMI, prednisolone, AEs
(Hao et al. 2025)	China	Single‐center retrospective cohort	77 (77/‐)	AChR‐Ab+	10 mg/kg weekly × 4; further cycles by clinical evaluation	12 weeks	MG‐ADL, MG‐ADL responders, AChR‐Ab+ responders, CMI, prednisolone, AEs
(Luo et al. 2024)	China	Multicenter real‐world cohort	61 (61/‐)	Mixed (AChR‐Ab+ 56; MuSK‐Ab+ 4)	10 mg/kg × 4 per cycle	12 weeks	MG‐ADL responders, AChR‐Ab+ and MuSK‐Ab+ responders, CMI, prednisolone, AEs
(Pane et al. 2024)	Italy	Multicenter retrospective cohort	31 (31/‐)^b^	Mixed (AChR‐Ab+ 22; MuSK‐Ab+ 3)	10 mg/kg IV weekly × 4 per cycle	Up to 48 weeks	MG‐ADL, QMG, MG‐ADL and QMG responders, AChR‐Ab+ and MuSK‐Ab+ responders, AEs
(Suzuki et al. 2024)	Japan	Registry‐based multicenter cohort	36 (36/‐)	Mixed (AChR‐Ab+ 19; MuSK‐Ab+ 6; seronegative 11)	10 mg/kg IV weekly × 4 per cycle; mean 2.4 cycles	26 weeks	MG‐ADL responders, AChR‐Ab+ and MuSK‐Ab+ responders, CMI, AEs
(Wang et al. 2024)	China	Prospective cohort	14 (14/‐)	AChR‐Ab+	10 mg/kg IV weekly × 4 (single cycle)	8 weeks	MG‐ADL, QMG, MG‐ADL and QMG responders, AChR‐Ab+ responders, CMI, AEs
Tier 3: Case series (single‐arm meta‐analysis)							
(Horiuchi et al. 2024)	Japan	Retrospective case series	16 (16/‐)	Double‐seronegative	10 mg/kg IV; two fixed cycles, then flexible	∼36 weeks	MG‐ADL, prednisolone, AEs
(Nomura et al. 2024)	Japan	Single‐center retrospective case series	16 (16/‐)	Mixed (AChR‐Ab+ 15; MuSK‐Ab+ 1)	10 mg/kg IV weekly × 4 (first cycle assessed)	Up to 13 months	QMG responders, AChR‐Ab+ and MuSK‐Ab+ responders, AEs
(Silvestri 2025)	USA	Single‐center retrospective case series	19 (19/‐)	AChR‐Ab+	10 mg/kg IV; three fixed cycles, then individualized	6–29 months	MG‐ADL, MG‐ADL responders, AChR‐Ab+ responders, CMI, prednisolone, AEs
Total: 11 studies			480 (385 efgartigimod/95 placebo)				

*Note*: Study aims, full eligibility criteria, and authors’ conclusions are provided in Table S2.

Abbreviations: AChR‐Ab+, acetylcholine receptor antibody‐positive; AEs, adverse events; CMI, clinically meaningful improvement; IV, intravenous; MuSK‐Ab+, muscle‐specific kinase antibody‐positive; RCT, randomized controlled trial.

^a^Continuous MG‐ADL and QMG outcomes from ADAPT refer to the AChR‐Ab+ population (65/64).

^b^The eculizumab arm of Pane et al. (*n* = 32) was not an eligible comparator and was not analyzed; total enrolment across the 11 studies was therefore 512.

### 2.9 Certainty of Evidence

The certainty of evidence for each outcome was rated using the GRADE approach (Guyatt et al. 2008), considering risk of bias, inconsistency, indirectness, imprecision, and publication bias. Evidence from RCTs started as high certainty and evidence from uncontrolled observational studies as low certainty. Imprecision was judged by whether the total sample size met the optimal information size and whether the 95% CI included both no effect and a clinically important effect (2 points for MG‐ADL and 3 points for QMG). For the randomized comparisons, inconsistency could not be assessed because only two trials were available. Both trials were sponsored by the manufacturer and shared the same first author; we considered this under publication bias but did not downgrade further, because both trials were published in full, and we instead report the absence of independent replication as a limitation (Section 4.3). Results are summarized in the summary of findings table (Section 3.7).

## 3 Results

### 3.1 Literature Search

We found 677 documents after performing a thorough literature search across three databases (PubMed, Scopus, and Web of Science). Title and abstract screening was performed on 452 documents after 225 duplicates were eliminated. For failing to meet the inclusion requirements, 436 of these were eliminated. Eight full‐text articles were eliminated during full‐text screening out of the 16 full‐text articles that were evaluated for eligibility. Three records were also manually obtained. Eleven studies in all met the criteria needed for inclusion and were incorporated into the meta‐analysis (Figure 1).

**FIGURE 1:**
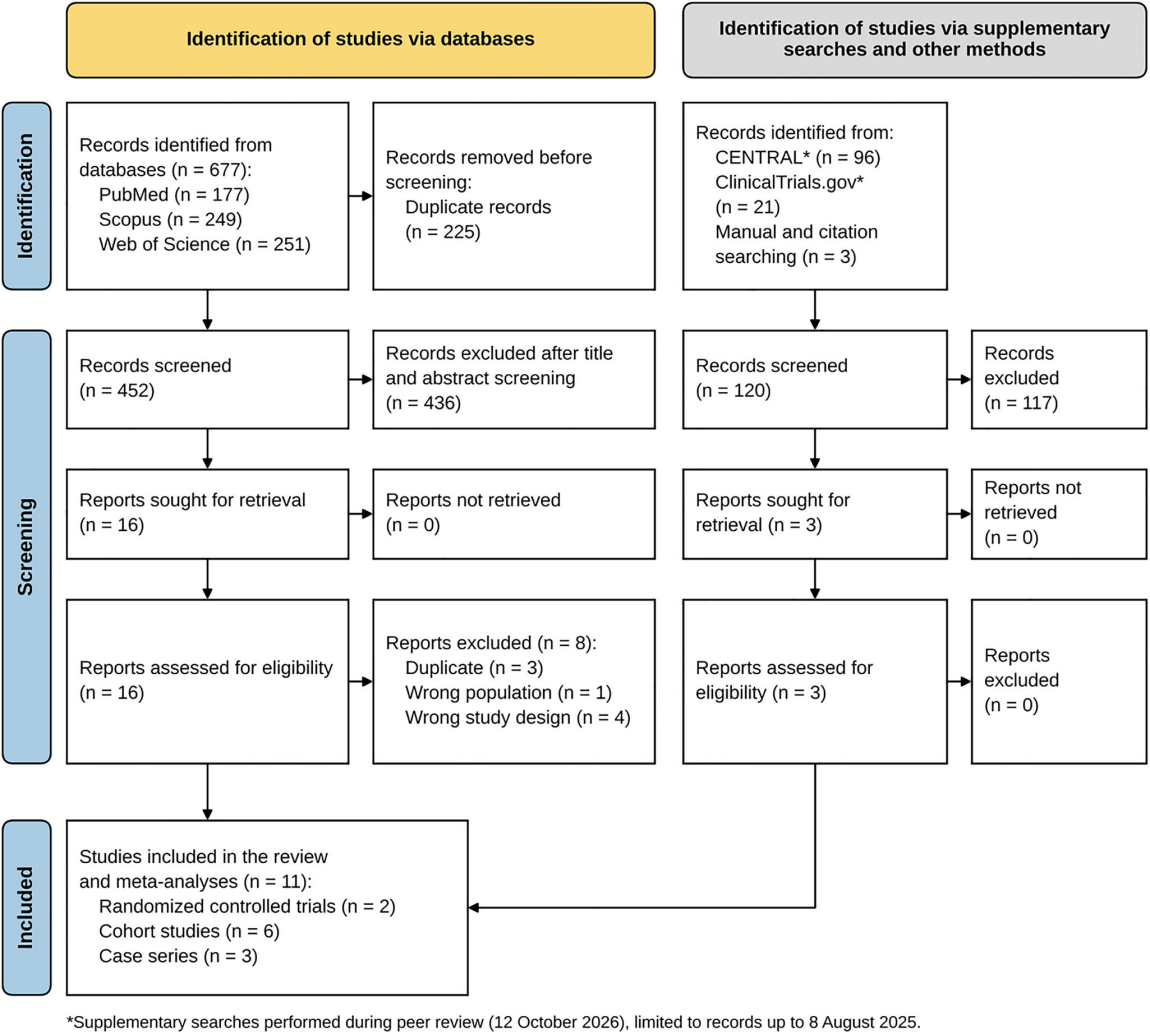
PRISMA 2020 flow diagram showing the study selection process for the systematic review and meta‐analysis. CENTRAL and ClinicalTrials.gov were searched during peer review (October 12, 2026), limited to records up to August 8, 2025, the end date of the original search; none of the 117 records retrieved met the eligibility criteria (Table S1). Layout based on the PRISMA 2020 template (Page et al. 2021).

The supplementary searches of CENTRAL and ClinicalTrials.gov, limited to records up to August 8, 2025, retrieved 96 and 21 records, respectively (117 in total). After these records were checked against the original search results and screened, none met the eligibility criteria, and no additional study was included (Figure 1; Table S1). Previous systematic reviews that searched CENTRAL or ClinicalTrials.gov likewise identified no placebo‐controlled trial of efgartigimod in gMG other than the two included in this review (Zhong et al. 2024; Cheng et al. 2025). The included studies and all analyses were therefore unchanged.

### 3.2 Study Characteristics

Eleven studies were included: two placebo‐controlled RCTs (Tier 1), six cohort studies (Tier 2), and three case series (Tier 3) (Table 1). These studies enrolled 512 participants. The 32 eculizumab‐treated patients in the study by Pane et al. (2024) did not meet the eligibility criteria for the intervention or comparator and were not included in any analysis. The review population therefore comprised 480 participants: 96 efgartigimod‐treated and 95 placebo‐treated participants in the two RCTs, and 289 efgartigimod‐treated patients in the nine observational studies. Study size ranged from 14 to 167 participants. Because not every study reported every outcome, the number of participants contributing to each analysis was smaller than 480; the analyzable sample size for each outcome is reported in the corresponding forest plots and in the summary of findings (Section 3.7). The studies included in this review were published between 2019 and 2025. They covered a narrow range of geographical distribution as China, Italy, and Japan were the most represented countries. A summary of the characteristics of the included studies detailed in Table 1 and baseline characteristics of the population reported in Table 2; study aims, full eligibility criteria, and authors’ conclusions are provided in Table S2.

**TABLE 2:** Baseline characteristics of included studies.

Study ID	Arm	Sample size	Age, mean (SD)	Male, *N* (%)	Disease duration, Mean (SD)	Prior thymectomy, *N* (%)	Baseline MG‐ADL, Mean (SD)	Baseline QMG, Mean (SD)	Prednisone dose, Mean (SD)	AChR‐Ab+, *N* (%)	MuSK‐Ab+, *N* (%)
(Frangiamore et al. 2024)	Efgartigimod	19	52.6 (11.9)	6 (31.6%)	9.7 (7.3)	7 (36.8%)	8.2 (3.1)	11.6 (4.8)	36.3 (14.7)	8 (42.1%)	4 (21%)
(Hao et al. 2025)	Efgartigimod	77	56.1 (15.2)	46 (59.7%)	3.6 (3.8)	21 (27.3%)	7.7 (3.6)	NR	26.3 (13.3)	77 (100%)	0 (0%)
(Horiuchi et al. 2024)	Efgartigimod	16	54.1 (11.7)	3 (18.8%)	NR	1 (6.3%)	9.2 (3.9)	NR	5.4 (2.19)	0 (0%)	0 (0%)
(Howard et al. 2019)	Efgartigimod	12	55.3 (13.6)	5 (41.7%)	8.2 (9)	5 (41.7%)	8 (3)	14.5 (6.3)	NR	12 (100%)	0 (0%)
	Placebo	12	43.5 (19.3)	4 (33.3%)	13.3 (11.2)	7 (58.3%)	8 (2.2)	11.8 (5.4)	NR	12 (100%)	0 (0%)
(Howard et al. 2021)	Efgartigimod	84	45.9 (14.4)	21 (25.0%)	10.1 (9)	59 (70.2%)	9.2 (2.6)	16.2 (5)	NR	65 (77.4%)	3 (3.6%)
	Placebo	83	48.2 (15)	28 (33.7%)	8.8 (7.6)	36 (43.4%)	8.8 (2.3)	15.5 (4.6)	NR	64 (77.1%)	3 (3.6%)
(Luo et al. 2024)	Efgartigimod	61	55.3 (16.4)	18 (29.5%)	5.1 (8.2)	28 (45.9%)	10.5 (5.2)	NR	28.3 (23)	56 (91.8%)	4 (6.6%)
(Nomura et al. 2024)	Efgartigimod	16	40.4 (13.2)	5 (31.3%)	14.3 (11.6)	12 (75.0%)	6.6 (NR)	13.8 (NR)	15.9 (NR)	15 (93.8%)	1 (6.3%)
(Pane et al. 2024)	Eculizumab	32	60.6 (15.5)	14 (43.8%)	10.8 (9.4)	15 (46.9%)	11 (3.4)	17.8 (6.1)	20.3 (18.7)	32 (100%)	0 (0%)
	Efgartigimod	31	51.9 (14.4)	9 (29.0%)	12.6 (9.6)	15 (48.4%)	9.9 (4.2)	15.3 (5.2)	12.4 (16.4)	22 (71.0%)	3 (9.7%)
Silvestri (2025)	Efgartigimod	19	52.7 (20.4)	11 (57.9%)	5.6 (3.3)	7 (36.8%)	8.3 (3.1)	NR	20 (20.7)	19 (100%)	0 (0%)
(Suzuki et al. 2024)	Efgartigimod	36	53 (14.7)	11 (31%)	12.9 (14.7)	16 (44.4%)	10.5 (4.3)	NR	12.6 (1.5)	19 (53%)	6 (17%)
(Wang et al. 2024)	Efgartigimod	14	57.7 (13.3)	7 (50%)	6.2 (5)	8 (57.1%)	10.86 (3.32)	20 (5.02)	NR	14 (100%)	0 (0%)

### 3.3 Quality Assessment

We evaluated both the two RCTs, Howard et al. (2021) and Howard et al. (2019), were deemed to have low risk of bias in all the domains (Figure 2). This high quality was because of their good methodologies like right randomization with allocation concealment, proper blinding of participants and outcome assessors, low and balanced rates of attrition, and pre‐specified analysis plans. Among the six non‐randomized cohort studies, the quality ranged from fair to good. Pane et al. (2024), Luo et al. (2024), Wang et al. (2024), and Frangiamore et al. (2024) were rated as good quality with NOS scores of 7 or 8 out of 9. Pane et al.'s (2024) study was unique in having a non‐randomized active comparator group, increasing its comparability domain. The two remaining studies, Hao et al. (2025) and Suzuki et al. (2024), were assessed as fair quality. The overriding limitation in most of these cohort studies was the absence of a contemporaneous comparator group that is directly comparable to the exposed group, which poses an additional risk of selection bias and confounding, and thus the ability to draw causal inferences (Table 3). All the three series of cases, Silvestri (2025), Horiuchi et al. (2024), and Nomura et al. (2024), were reported to have followed the proper procedures for their study design in accordance with the JBI checklist. However, since they lack a comparator group by nature, they would qualify as high‐risk bias (Table 4).

**FIGURE 2:**
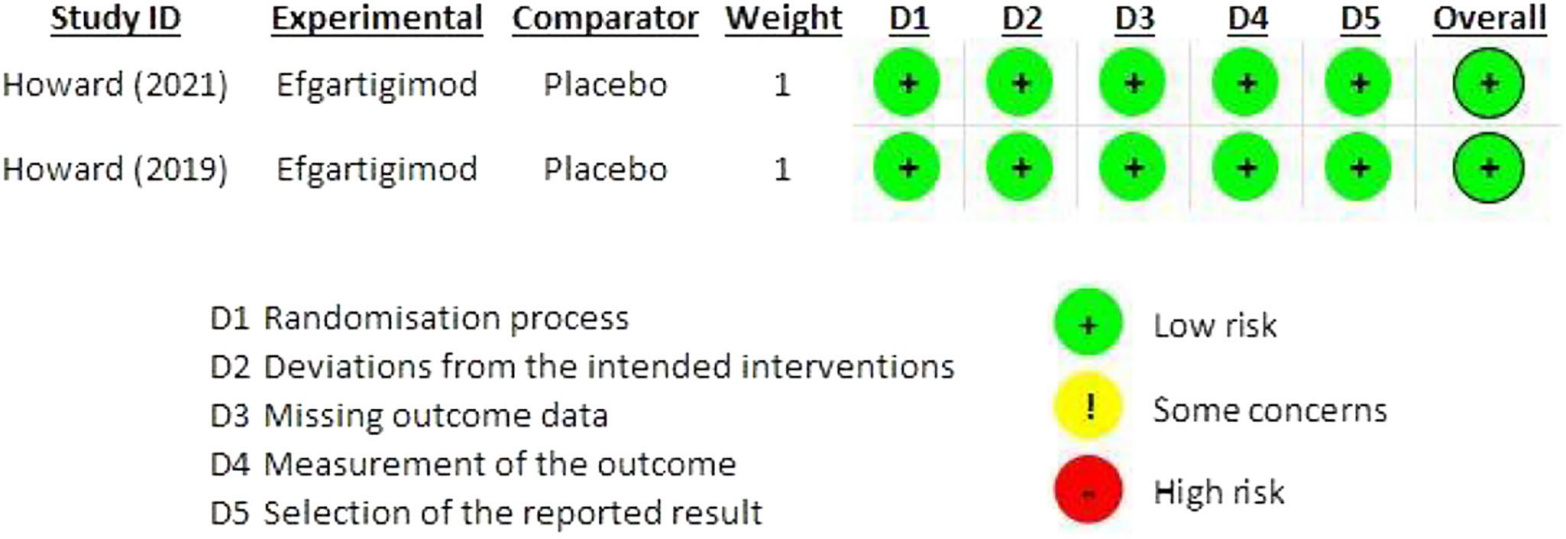
Risk of bias assessment of the included randomized controlled trials using the Cochrane RoB 2 tool.

**TABLE 3:** Newcastle–Ottawa Scale for quality assessment of cohort studies.

Study ID	Representativeness of the exposed cohort	Selection of the nonexposed cohort	Ascertainment of exposure	Demonstration that outcome of interest was not present at start of study	Comparability of cohorts on the basis of the design or analysis	Assessment of outcome	Was follow‐up long enough for outcomes to occur	Adequacy of follow‐up of cohorts
Pane et al. (2024)	★	★	★	★	★	★	★	★
Luo et al. (2024)	★	★	★	★	—	★	★	★
Wang et al. (2024)	★	★	★	★	—	★	★	★
Frangiamore et al. (2024)	★	★	★	★	—	★	★	★
Hao et al. (2025)	★	★	★	★	—	★	★	—
Suzuki et al. (2024)	★	★	★	★	—	★	★	—

*Note*: A star (★) indicates that the criterion was met and a dash (‐) indicates that it was not; maximum score = 9. Studies scoring ≥ 7 were rated good quality and those scoring 6 fair quality.

**TABLE 4:** Joanna Briggs Institute (JBI) Critical Appraisal Checklist for quality assessment of case series studies.

Criteria	Silvestri (2025)	Horiuchi et al. (2024)	Nomura et al. (2024)
1. Were the inclusion criteria clear?	Yes	Yes	Yes
2. Were the patients and setting similar to my population?	Yes	Yes	Yes
3. Was the exposure measured in a valid and reliable way?	Yes	Yes	Yes
4. Were the outcomes measured in a valid and reliable way?	Yes	Yes	Yes
5. Were there clear criteria for who was included (e.g., consecutive)?	Yes	Yes	Yes
6. Was follow‐up complete and long enough?	Yes	Yes	Yes
7. Are the results clearly and detailedly reported?	Yes	Yes	Yes
8. Was the statistical analysis appropriate?	Yes	Yes	Yes

### 3.4 Randomized Controlled Evidence: Two‐Arm Meta‐Analysis (Tier 1)

Both RCTs were sponsored by the manufacturer and shared the same first author, but they enrolled separately recruited patient cohorts at different times (December 2016–July 2017 and September 2018–November 2019; Table S2). The phase 3 ADAPT trial contributed 70.9%–91.5% of the statistical weight in each two‐arm analysis (Figures 3, 4, 5), so the pooled estimates largely reflect its results. With only two trials, the *I* ^2^ values of 0% reported below cannot be taken as evidence that the treatment effect is consistent across settings.

**FIGURE 3:**
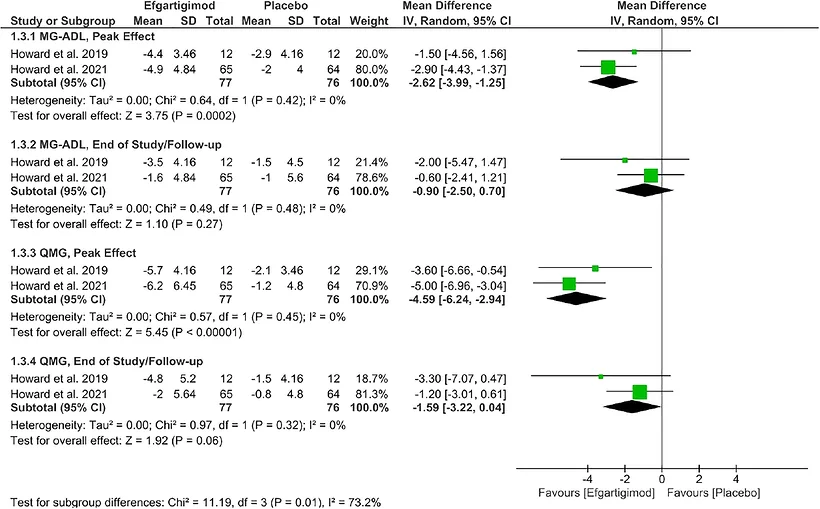
Forest plot of mean change in MG‐ADL and QMG scores with efgartigimod versus placebo in the randomized controlled trials, at peak effect and at the end of follow‐up.

**FIGURE 4:**
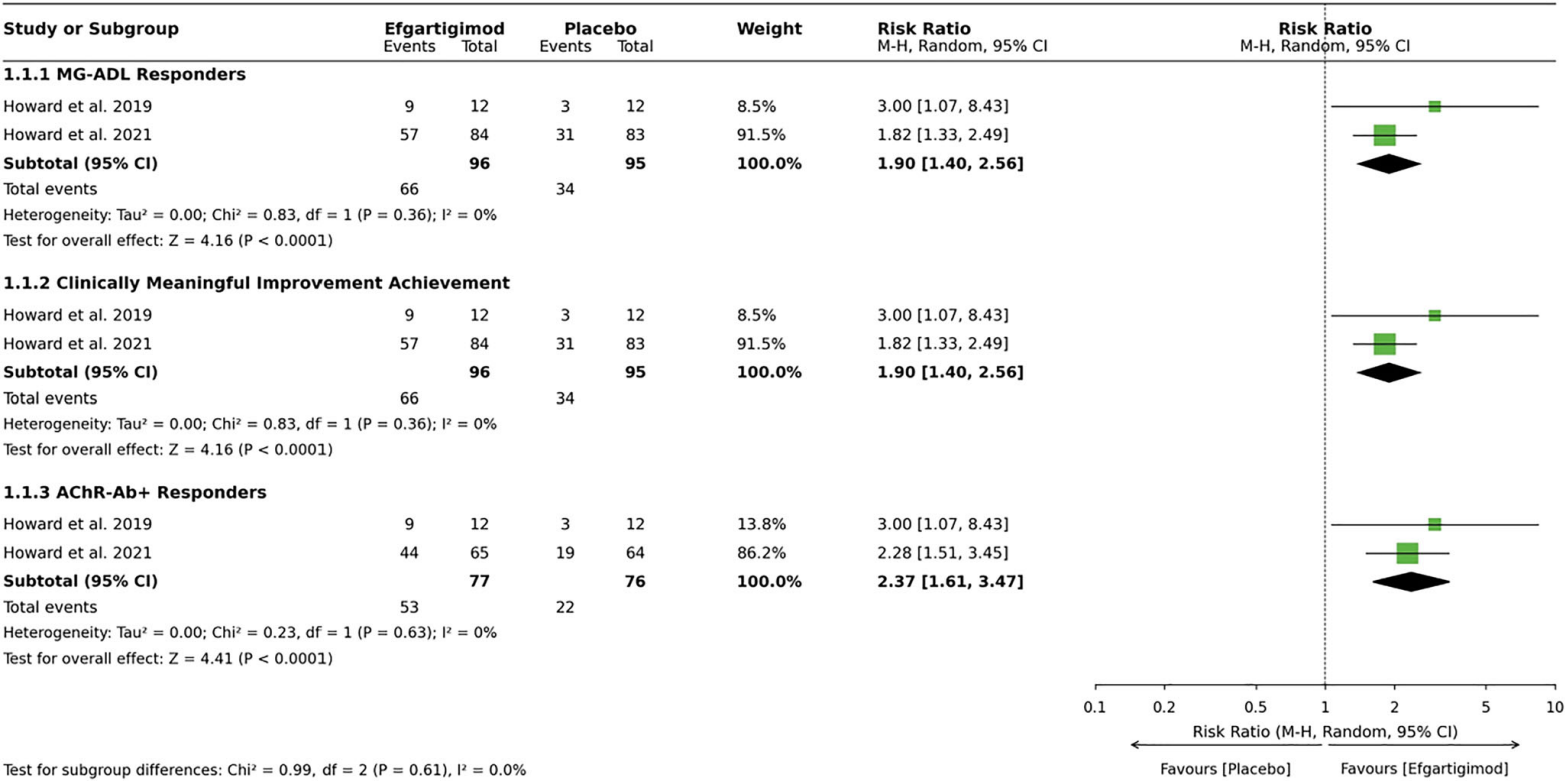
Forest plot of MG‐ADL responders, clinically meaningful improvement, and AChR‐Ab+ responders with efgartigimod versus placebo in the randomized controlled trials. Clinically meaningful improvement was defined using the MG‐ADL responder criterion in both trials and therefore shares the same event counts.

**FIGURE 5:**
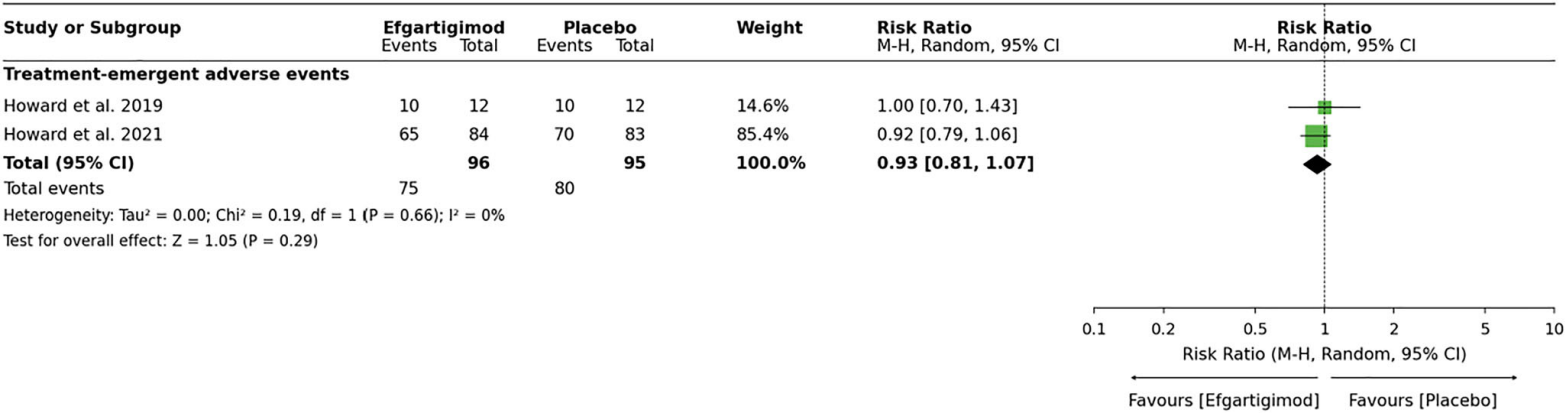
Forest plot of the incidence of adverse events with efgartigimod versus placebo in the randomized controlled trials.

### 3.5 Efficacy Outcomes

#### 3.5.1 MG‐ADL Improvement

A pooled analysis of the two RCTs (153 participants) found significant improvements with efgartigimod compared to placebo at peak effect (MD = −2.62, 95% CI: −3.99 to −1.25, *p* = 0.0002) with no heterogeneity (*I* ^2^ = 0%, *p* = 0.42). By follow‐up, the difference was no longer significant (MD = −0.90, 95% CI: −2.50 to 0.70, *p* = 0.27; *I* ^2^ = 0%, *p* = 0.48) (Figure 3).

#### 3.5.2 QMG Improvement

Efgartigimod demonstrated strong efficacy at the peak effect (MD = −4.59, 95% CI: −6.24 to −2.94, *p* < 0.00001; 153 participants), with no heterogeneity (*I* ^2^ = 0%, *p* = 0.45). The end‐of‐study data showed no sustained significance (MD = −1.59, 95% CI: −3.22 to 0.04, *p* = 0.06; *I* ^2^ = 0%, *p* = 0.32) (Figure 3).

#### 3.5.3 MG‐ADL Responders and Clinically Meaningful Improvement

Efgartigimod was associated with a significantly higher proportion of MG‐ADL responders than placebo (66/96 [68.8%] vs. 34/95 [35.8%]; RR = 1.90, 95% CI: 1.40–2.56, *p* < 0.0001; 191 participants), with no heterogeneity (*I* ^2^ = 0%, *p* = 0.36) (Figure 4). In both RCTs, clinically meaningful improvement was operationalized using the MG‐ADL responder criterion (a sustained reduction of ≥ 2 points in the MG‐ADL score). This outcome was therefore based on the same event counts and yielded an identical estimate (RR = 1.90, 95% CI: 1.40–2.56), and it is reported jointly with MG‐ADL responders rather than as an independent finding.

#### 3.5.4 AChR‐Ab+ Responders

In AChR‐Ab+ participants, the proportion of MG‐ADL responders was higher with efgartigimod than with placebo (53/77 [68.8%] vs. 22/76 [28.9%]; RR = 2.37, 95% CI: 1.61–3.47, *p* < 0.0001; 153 participants), with no heterogeneity (*I* ^2^ = 0%, *p* = 0.63) (Figure 4).

### 3.6 Safety Outcomes

#### 3.6.1 Adverse Events

In the two RCTs (191 participants), adverse events occurred in 75/96 (78.1%) efgartigimod‐treated and 80/95 (84.2%) placebo‐treated participants. The pooled incidence did not differ significantly between groups (RR = 0.93, 95% CI: 0.81–1.07, *p* = 0.29), with no heterogeneity (*I* ^2^ = 0%, *p* = 0.66) (Figure 5).

#### 3.6.2 Sensitivity Analyses of Randomized Evidence

Because the estimated between‐trial variance was zero for every two‐arm outcome (tau^2^ = 0), although it cannot be estimated reliably from two trials, fixed‐effect models gave materially identical estimates (for example, MG‐ADL responders RR = 1.92, 95% CI: 1.42–2.60; adverse events RR = 0.93, 95% CI: 0.81–1.06). Restricting the analyses to the phase 3 ADAPT trial (Howard et al. 2021) did not change any conclusion: MG‐ADL at peak effect, MD = −2.90 (95% CI: −4.43 to −1.37); QMG at peak effect, MD = −5.00 (95% CI: −6.96 to −3.04); MG‐ADL responders, RR = 1.82 (95% CI: 1.33–2.49); AChR‐Ab+ responders, RR = 2.28 (95% CI: 1.51–3.45); and adverse events, RR = 0.92 (95% CI: 0.79–1.06). With only two trials, subgroup analysis and meta‐regression were not feasible.

### 3.7 Uncontrolled Real‐World and Case‐Series Evidence: Single‐Arm Meta‐analysis (Tiers 2 and 3)

The following analyses pooled within‐group changes or proportions from studies without a placebo or untreated comparator. They describe outcomes observed in efgartigimod‐treated patients in routine practice and cannot separate treatment effects from regression to the mean, natural disease fluctuation, or concomitant therapy; they are therefore presented as supportive of, rather than equivalent to, the randomized evidence. The corresponding forest plots are provided as Figures S1–S9.

### 3.8 Efficacy Outcomes

#### 3.8.1 MG‐ADL Improvement

Six studies (173 patients) found significant reductions in scores (pooled mean change = −4.97, 95% CI: −6.44 to −3.50; 95% PI: −10.05 to 0.11), with considerable heterogeneity (*I* ^2^ = 86.7%, tau = 1.67 points, *p* < 0.0001) (Figure S1). Every study reported a reduction whose 95% CI excluded zero; study‐specific changes ranged from −1.80 points in the double‐seronegative cohort (Horiuchi et al. 2024) to −6.90 points (Hao et al. 2025).

#### 3.8.2 QMG Improvement

Three studies (64 patients) demonstrated significant reductions (pooled mean change = −4.83, 95% CI: −6.69 to −2.97; 95% PI: −24.83 to 15.17, uninformative with three studies) with moderate heterogeneity (*I* ^2^ = 55.9%, *p* = 0.10) (Figure S2).

#### 3.8.3 MG‐ADL Responders

Seven studies (254 patients) showed high treatment efficacy (proportion = 0.947, 95% CI: 0.813–0.986; 95% PI: 0.190–0.999), but with substantial heterogeneity (*I* ^2^ = 74.0%, *p* = 0.0008) (Figure S3); study‐specific proportions ranged from 0.618 (Suzuki et al. 2024) to 1.000 (Frangiamore et al. 2024; Silvestri 2025).

#### 3.8.4 QMG Responders

Four studies (80 patients) found increasing response rates (proportion = 0.744, 95% CI: 0.380–0.932; 95% PI: 0.003–1.000, uninformative with four studies) and substantial heterogeneity (*I* ^2^ = 65.6%, *p* = 0.0334) (Figure S4).

#### 3.8.5 AChR+ Responders

Pooled analysis of eight studies (228 patients) demonstrated high response rates among AChR‐Ab+ patients (proportion = 0.935, 95% CI: 0.744–0.986; 95% PI: 0.091–1.000) with moderate heterogeneity (*I* ^2^ = 48.5%, *p* = 0.0591) (Figure S5).

#### 3.8.6 MuSK+ Responders

Pooled data from five studies (18 patients) revealed positive responses in MuSK‐Ab+ patients, although the estimate was based on very few patients (proportion = 0.781, 95% CI: 0.499–0.927; 95% PI: 0.257–0.974) with no heterogeneity (*I* ^2^ = 0.0%, *p* = 0.8818) (Figure S6).

#### 3.8.7 Clinically Meaningful Improvement

Six of the studies (223 patients) reported high rates of improvement (proportion = 0.884, 95% CI: 0.714–0.959; 95% PI: 0.148–0.997), but with considerable heterogeneity (*I* ^2^ = 81.1%, *p* < 0.0001) (Figure S7); study‐specific proportions ranged from 0.618 (Suzuki et al. 2024) to 0.987 (Hao et al. 2025).

#### 3.8.8 Prednisolone Dosage Reduction

Five studies reported a significant reduction (mean change = −10.81 mg/day, 95% CI: −16.31 to −5.31; 95% PI: −31.37 to 9.75) with considerable heterogeneity (*I* ^2^ = 98.8%, tau = 5.82 mg/day, *p* < 0.0001) (Figure S8). The heterogeneity concerned the magnitude rather than the direction of change: all five studies reported a reduction whose 95% CI excluded zero, ranging from −1.30 mg/day in the double‐seronegative cohort, whose mean baseline dose was 5.4 mg/day (Horiuchi et al. 2024), to −16.90 mg/day (Silvestri 2025). Relative to the mean baseline doses reported by each study (Table 2), these reductions corresponded to approximately 24%–85% of the baseline dose. The pooled estimate should therefore be interpreted as an average across settings with different baseline doses rather than as a single expected reduction.

### 3.9 Safety Outcomes

#### 3.9.1 Adverse Events

Nine studies (286 patients) reported moderate incidence rates (proportion = 0.205, 95% CI: 0.104–0.363; 95% PI: 0.016–0.799) with considerable heterogeneity (*I* ^2^ = 81.3%, *p* < 0.0001) (Figure S9). Study‐specific proportions ranged from 0.039 (Hao et al. 2025) to 0.684 (Frangiamore et al. 2024), which probably reflects differences in the definition and ascertainment of adverse events across studies.

### 3.10 Sensitivity, Influence, and Subgroup Analyses of Single‐Arm Outcomes

Leave‐one‐out analyses showed that no single study altered the direction or statistical significance of any pooled single‐arm estimate (Table S3). Pooled estimates ranged from −4.53 to −5.74 points for MG‐ADL change, −4.14 to −5.37 points for QMG change, 0.927 to 0.963 for MG‐ADL responders, 0.595 to 0.816 for QMG responders, 0.881 to 0.956 for AChR‐Ab+ responders, 0.714 to 0.857 for MuSK‐Ab+ responders, 0.827 to 0.916 for clinically meaningful improvement, −9.85 to −13.60 mg/day for prednisolone dose, and 0.165 to 0.250 for adverse events.

The influence analyses identified the main sources of statistical heterogeneity. Omitting Horiuchi et al. (2024), the only study restricted to double‐seronegative patients, increased the pooled MG‐ADL reduction to −5.74 points (95% CI: −6.67 to −4.81) and reduced *I* ^2^ from 86.7% to 60.2%. For prednisolone dose, omitting the same study, whose patients received low baseline doses (mean 5.4 mg/day), yielded a reduction of −13.60 mg/day (95% CI: −14.59 to −12.61) with no residual heterogeneity (*I* ^2^ = 0%). Omitting Suzuki et al. (2024) reduced heterogeneity for MG‐ADL responders (*I* ^2^ from 74.0% to 51.5%), and omitting Frangiamore et al. (2024) removed heterogeneity for QMG responders (*I* ^2^ = 0%; pooled proportion 0.816, 95% CI: 0.570–0.937).

In subgroup analyses by study design, pooled estimates from cohort studies and case series did not differ significantly for MG‐ADL change (−5.59 vs. −3.97 points; *p* for subgroup difference = 0.47), AChR‐Ab+ responders (0.933 vs. 0.941; *p* = 0.95), prednisolone dose (−13.53 vs. −8.35 mg/day; *p* = 0.51), or adverse events (0.169 vs. 0.294; *p* = 0.28). Restricting the analyses to good‐quality cohort studies (NOS ≥ 7) produced consistent estimates, for example, −4.96 points (95% CI: −5.85 to −4.07; *I* ^2^ = 0%) for MG‐ADL change and 0.930 (95% CI: 0.806–0.977) for MG‐ADL responders (Table S4).

The 95% PIs were wide for every single‐arm outcome (Section 3.5; Table 5). For MG‐ADL change, the interval (−10.05 to 0.11 points) indicates that a new study in a similar population could observe anything from a large improvement to almost no change, and for prednisolone dose the interval (−31.37 to 9.75 mg/day) was uninformative. For responder outcomes, the intervals were further widened because several studies reported response proportions of 100%, which inflates the between‐study variance on the logit scale. These findings confirm that the single‐arm pooled estimates describe average outcomes across heterogeneous settings and should not be interpreted as precise estimates of a treatment effect.

**TABLE 5:** Summary of findings and certainty of evidence (GRADE) for all outcomes, with the number of studies and participants contributing to each analysis.

Outcome	Studies (participants)	Effect (95% CI); 95% PI for single‐arm outcomes	Anticipated absolute effect	Certainty (GRADE)	Reasons for downgrading
Randomized evidence: efgartigimod vs placebo					
MG‐ADL change, peak effect	2 RCTs (153)	MD −2.62 (−3.99 to −1.25)	—	Moderate	Imprecision (−1): sample below optimal information size
MG‐ADL change, end of follow‐up	2 RCTs (153)	MD −0.90 (−2.50 to 0.70)	—	Low	Imprecision (−2): CI includes no effect and the 2‐point MCID
QMG change, peak effect	2 RCTs (153)	MD −4.59 (−6.24 to −2.94)	—	Moderate	Imprecision (−1)
QMG change, end of follow‐up	2 RCTs (153)	MD −1.59 (−3.22 to 0.04)	—	Low	Imprecision (−2): CI includes no effect and the 3‐point MCID
MG‐ADL responders (including CMI)	2 RCTs (191)	RR 1.90 (1.40–2.56)	358 vs. 680 per 1000; 322 more per 1000 (143–558 more)	Moderate	Imprecision (−1)
AChR‐Ab+ responders	2 RCTs (153)	RR 2.37 (1.61–3.47)	289 vs. 686 per 1000; 397 more per 1000 (at least 177 more)	Moderate	Imprecision (−1)
Adverse events	2 RCTs (191)	RR 0.93 (0.81–1.07)	842 vs. 783 per 1000; 59 fewer per 1000 (160 fewer to 59 more)	Moderate	Imprecision (−1): CI includes no difference and small harm or benefit
Uncontrolled real‐world evidence: single‐arm estimates (no comparator)					
MG‐ADL change from baseline	6 (173)	Mean change −4.97 (−6.44 to −3.50); PI −10.05 to 0.11	—	Very low	No comparator; risk of bias; inconsistency (*I* ^2^ = 86.7%)
QMG change from baseline	3 (64)	Mean change −4.83 (−6.69 to −2.97); PI −24.83 to 15.17	—	Very low	No comparator; risk of bias; imprecision
MG‐ADL responders	7 (254)	Proportion 0.947 (0.813–0.986); PI 0.190–0.999	—	Very low	No comparator; inconsistency (*I* ^2^ = 74.0%)
QMG responders	4 (80)	Proportion 0.744 (0.380–0.932); PI 0.003–1.000	—	Very low	No comparator; inconsistency; imprecision
AChR‐Ab+ responders	8 (228)	Proportion 0.935 (0.744–0.986); PI 0.091–1.000	—	Very low	No comparator; inconsistency (*I* ^2^ = 48.5%)
MuSK‐Ab+ responders	5 (18)	Proportion 0.781 (0.499–0.927); PI 0.257–0.974	—	Very low	No comparator; very serious imprecision
Clinically meaningful improvement	6 (223)	Proportion 0.884 (0.714–0.959); PI 0.148–0.997	—	Very low	No comparator; inconsistency (*I* ^2^ = 81.1%)
Prednisolone dose change	5 (NR^a^)	Mean change −10.81 mg/day (−16.31 to −5.31); PI −31.37 to 9.75	—	Very low	No comparator; inconsistency (*I* ^2^ = 98.8%)
Adverse events	9 (286)	Proportion 0.205 (0.104–0.363); PI 0.016–0.799	—	Very low	No comparator; inconsistency (*I* ^2^ = 81.3%); variable AE definitions

*Note*: Anticipated absolute effects are based on the pooled placebo‐group risk in the RCTs. GRADE: evidence from RCTs starts at high certainty and evidence from uncontrolled studies at low certainty. Moderate, the true effect is probably close to the estimate; low, the true effect may be substantially different; very low, the true effect is probably substantially different.

Abbreviations: CI, confidence interval; CMI, clinically meaningful improvement; MCID, minimal clinically important difference; MD, mean difference; PI, 95% prediction interval; RR, risk ratio.

^a^ Study‐level numbers of corticosteroid‐treated patients were not consistently reported. With only two randomized trials, inconsistency could not be assessed; both trials were sponsored by the manufacturer and the phase 3 ADAPT trial contributed 70.9%–91.5% of the weight in each comparison, so the randomized estimates do not represent independent replication. Prediction intervals were calculated with a *t* distribution with *k* − 2 degrees of freedom and are uninformative when few studies contribute.

### 3.11 Certainty of Evidence

Certainty of evidence was moderate for MG‐ADL and QMG improvement at peak effect, MG‐ADL responders, AChR‐Ab+ responders, and adverse events in the randomized comparison; these outcomes were downgraded once for imprecision because the total sample size was below the optimal information size. Certainty was low for MG‐ADL and QMG change at the end of follow‐up, which were downgraded twice for imprecision because the 95% CIs included both no effect and a clinically important benefit. All single‐arm outcomes were rated as very low certainty because of the absence of a comparator group, risk of bias, and, for most outcomes, substantial inconsistency or imprecision (Table 5).

## 4 Discussion

### 4.1 Summary of Findings

In this meta‐analysis of 11 studies that included 480 patients with gMG, we examined the efficacy and safety of efgartigimod, analyzing two placebo‐controlled RCTs separately from nine uncontrolled real‐world studies. The randomized evidence showed that efgartigimod significantly improved major clinical outcomes by lowering MG‐ADL and QMG in the short term at peak effect, but there was no sustained impact at follow‐up. The proportion of MG‐ADL responders was almost twice as high with efgartigimod as with placebo, including among AChR‐Ab+ patients, with moderate certainty of evidence. The incidence of adverse events did not differ significantly between efgartigimod and placebo. Furthermore, single‐arm analyses of real‐world studies, which provide very low‐certainty evidence, showed improved effectiveness outcomes, with significant responder rates in both AChR+ and MuSK+ patients. Also, we reported a reduction in prednisolone dosage while moderate adverse event rates were present. The randomized estimates were unchanged in fixed‐effect models and when restricted to the ADAPT trial, and no single study altered the direction or significance of any single‐arm estimate. However, the randomized evidence derives from two manufacturer‐sponsored trials dominated by ADAPT, and the single‐arm estimates showed considerable heterogeneity with wide PIs, so the latter should be interpreted descriptively.

### 4.2 Interpretation of Findings

The treatment of individuals with gMG who have social challenges and an inadequate quality of life has received a lot of attention lately (Schneider‐Gold et al. 2019; Tran et al. 2021). A novel therapeutic approach is needed to address the adverse condition with side effects that corticosteroid and other immunosuppressive medication treatment can cause. A 68% response rate with a wide range of improvement was observed by the ADAPT group. Four patients had a substantial response, displaying a ≥ 9‐point reduction in the MG‐ADL score, and six patients had a narrow response, presenting a 2‐point or 3‐point reduction in the MG‐ADL score, out of the 21 respondents in these studies (Howard et al. 2019). Since it was stated that clinical improvement lasting at least 6 weeks was often attained, some patients expressed dissatisfaction that the duration of efgartigimod's efficacy was less than anticipated.

Evidence from real‐world studies from countries like China, Japan, Italy, and others shed light on the long‐term safety and effectiveness of efgartigimod in the treatment of gMG in a variety of demographics. Suzuki et al. (2024) published the results of a multicenter research on 36 patients who were given efgartigimod. Significant improvements were noted in the MG‐ADL and MG‐QOL15‐*r* scores. The study came to the conclusion that efgartigimod is a practical and well‐tolerated medication for Japanese MG patients, and it validated its effectiveness across a number of MG subtypes. In a real‐world evaluation, Frangiamore et al. (2024) found that efgartigimod significantly improved clinical outcomes and reduced hospitalizations. Efgartigimod therapy was well tolerated, with no serious side effects noted throughout the 14‐month follow‐up period.

In another study, efgartigimod‐treated patients maintained a steady response despite being administered in treatment cycles compared to a continuous strategy for eculizumab (Pane et al. 2024). This study is the first to continuously report the mean MG‐ADL and QMG values of participants treated with efgartigimod. This was made achievable by administering the MG‐ADL weekly and administering QMG on a regular basis, in accordance with recent expert consensus recommendations for monitoring gMG patients (Meisel et al. 2024). According to Silvestri (2025), patients were able to lower the dosage and/or stop using their concurrent drugs, particularly corticosteroids, after completing around four cycles of efgartigimod treatment. These findings imply that treating gMG patients with efgartigimod may be one way to accomplish corticosteroid tapering.

Subgroup analysis from the ADAPT study indicate efgartigimod is efficacious in gMG patients, regardless of illness stage or treatment history (Howard et al. 2021). Wang et al. (2024) found that individuals with various disease durations, clinical scales, and baseline treatment regimens experienced significant improvements in MG‐ADL and QMG scores after a single cycle of efgartigimod. In the Nomura et al. (2024) study, 71% of patients experienced clinically significant improvements in QMG scores following the first cycle. In the ADAPT trial, 63% of patients in the efgartigimod group achieved QMG response in the first cycle (Howard et al. 2021). Patients in this cohort had similar sex, antibody status, and history of thymectomy to those in the ADAPT study.

According to available RCT's evidence, efgartigimod's clinical benefit was originally linked to the patients' decreased IgG levels but continued even after they reached their baseline levels (Howard et al. 2019, 2021). The evidence reported by Luo et al. (2024) found that efgartigimod had positive effects in gMG, regardless of antibody type. Notably, MuSK‐MG demonstrated a satisfactory reduction in MG‐ADL following therapy, supporting the ADAPT+ research and an Italian multicenter cohort investigation (Howard et al. 2021; Frangiamore et al. 2024). Efgartigimod was equally effective in eliminating pathogenic IgGs in IgG1, IgG3, and IgG4. At 1.3 weeks, 97% (58 of 61) of patients showed clinically significant improvement. By week 12, the MG‐ADL score had declined to 3.8 (Luo et al. 2024).

The percentage of patients in the Hao et al. (2025) study who experienced minimal symptom expression (MSE) following one cycle of efgartigimod treatment (69.7% vs. 45.9%) and clinical meaningful improvement (CMI) following one cycle of efgartigimod treatment (97.4% vs. 72%) was higher than that of the Luo et al. (2024) study. The exclusion of patients having MG crises from that study may be the cause of this disparity. Furthermore, the Hao et al. (2025) study population had a shorter time between the onset of their disease and the start of efgartigimod (3.6 years), which might improve their response to the medication. This suggests that early efgartigimod administration will be worthwhile to be investigated in order to improve outcomes for MG patients. In our pooled analysis of the two RCTs, the incidence of adverse events did not differ significantly between efgartigimod and placebo, and the reported events were predominantly mild to moderate, consistent with the favorable tolerability observed in the real‐world cohorts.

Our randomized estimates agree closely with those of earlier syntheses (Table S5). Cheng et al. (2025) reported RR of 1.85 (95% CI: 1.38–2.46) for response and 0.93 (95% CI: 0.81–1.07) for adverse events in their analysis of the same two RCTs, compared with 1.90 and 0.93 in our analysis; Saccà et al. (2023) reported a pooled QMG difference of −4.78 points for anti‐FcRn agents, similar to our peak‐effect estimate of −4.59 points; and Zhong et al. (2024) found that the QMG benefit of efgartigimod in ADAPT was no longer significant 4 weeks after the last dose, consistent with our end‐of‐follow‐up findings. This agreement is expected, because all of these analyses draw on the same trials, and it underscores that the comparative evidence for efgartigimod has not yet been independently replicated.

Our findings are consistent with previous meta‐analyses of randomized trials that found FcRn inhibitors, including efgartigimod, to be effective in AChR‐Ab+ gMG (Saccà et al. 2023; Li et al. 2024), and they extend this evidence in three clinically relevant respects. First, the benefit over placebo was clear at peak effect but was no longer statistically significant at the end of follow‐up. This pattern is consistent with the pharmacodynamics of FcRn blockade, in which IgG levels recover after a treatment cycle, and supports cyclical, individualized retreatment guided by symptom recurrence, as described in real‐world studies using flexible dosing (Frangiamore et al. 2024; Silvestri 2025). Second, real‐world data suggested responses in MuSK‐Ab+ patients and a clinically relevant corticosteroid‐sparing effect, two questions that the pivotal trials were not designed to answer. However, the MuSK‐Ab+ estimate was based on only 18 patients and all single‐arm estimates were of very low certainty, so these findings should be regarded as hypothesis‐generating. Third, the only study limited to double‐seronegative patients (Horiuchi et al. 2024) reported smaller improvements and was the main source of heterogeneity for MG‐ADL and corticosteroid outcomes, suggesting that serological status may modify the magnitude of response and should be considered when setting treatment expectations.

Clinical heterogeneity across the included studies was considerable and probably explains much of the statistical heterogeneity observed in the single‐arm analyses. Studies differed in antibody status (AChR‐Ab+ only, mixed serotypes, or double‐seronegative), disease severity and refractoriness, dosing schedule (a single fixed cycle, two fixed cycles, or individualized retreatment), timing of outcome assessment (from 8 weeks to more than 2 years), and responder definitions, which ranged from a sustained reduction of ≥ 2 points in MG‐ADL over consecutive weeks in the RCTs to a reduction at a single time point in several real‐world studies. These differences are the most plausible explanation for the variability in pooled estimates, and they support our decision to base comparative inferences on the randomized evidence alone.

Statistical heterogeneity in the single‐arm analyses was considerable for several outcomes, with *I* ^2^ values up to 98.8%. Such values preclude interpreting the pooled single‐arm estimates as a single common effect; we have therefore reported PIs and the range of study‐specific estimates alongside each pooled result and graded all single‐arm outcomes as very low certainty. Two features nevertheless aid interpretation. First, *I* ^2^ is a relative measure that approaches 100% when individual studies are precise, as in large cohorts reporting response proportions close to 1, even when absolute differences between studies are modest (Borenstein et al. 2017; Migliavaca et al. 2022); for MG‐ADL change, for example, the between‐study standard deviation (tau) was 1.67 points. Second, the heterogeneity concerned the magnitude rather than the direction of the observed changes: every study showed a reduction in MG‐ADL score and in corticosteroid dose, and the main sources of heterogeneity were identifiable, namely the double‐seronegative cohort for MG‐ADL and corticosteroid outcomes and differences in baseline corticosteroid dose. These analyses indicate what clinicians may observe in practice, but they cannot substitute for controlled comparisons.

### 4.3 Strengths, Limitations, and Recommendations

Strengths of this review include the separation of randomized and uncontrolled evidence into distinct analytic tiers, the evaluation of outcomes at both peak effect and the end of follow‐up, the inclusion of real‐world studies that address serotypes and corticosteroid‐sparing effects not examined in the pivotal trials, the use of sensitivity and influence analyses, the reporting of PIs, and the grading of the certainty of evidence with GRADE. The review was reported in accordance with the PRISMA statement.

It is essential to recognize the small sample sizes of the included research. Furthermore, some of the reported outcomes were heterogeneous as number of drug cycles and patient characteristics were different, which may limit how widely our findings may be applied. Larger, multicenter trials are particularly necessary to validate these findings across multiple patient demographics and healthcare settings. In this respect, ADAPT SERON (NCT06298552), a placebo‐controlled phase 3 trial in 119 patients with AChR‐Ab‐negative gMG, including MuSK‐Ab+, LRP4‐Ab+, and triple‐seronegative disease, reported a statistically significant improvement in MG‐ADL at day 29 (*p* = 0.0068) in topline results announced after our search end date (argenx 2025); its full publication will allow future updates of this review to include randomized evidence specific to these subgroups, ideally alongside trials conducted independently of the manufacturer. Consistent outcome definitions should be utilized in similar investigations to remove any potential biases or variability discovered in the current study. Several further limitations should be acknowledged. First, the review protocol was registered in PROSPERO retrospectively rather than before the literature search, which increases the potential for reporting bias; we have sought to mitigate this by reporting all analyzed outcomes and the complete search strategy, labeling all sensitivity, influence, and subgroup analyses as post hoc, and providing the extracted study‐level data (Table S6). Second, only two RCTs were available, both sponsored by the manufacturer and sharing the same first author, and ADAPT contributed most of the statistical weight; the comparative estimates therefore do not represent independent replication, and between‐trial heterogeneity could not be estimated. Moreover, both trials enrolled predominantly AChR‐Ab+ patients, so comparative evidence for MuSK‐Ab+ and seronegative disease is lacking. Third, nine of the 11 studies had no control group; single‐arm estimates therefore cannot be interpreted as treatment effects and were rated as very low certainty; several of these analyses also showed considerable heterogeneity and wide PIs. Fourth, fewer than 10 studies contributed to each outcome, which precluded meta‐regression and formal assessment of publication bias. Fifth, the search was limited to records available up to August 2025, so studies published afterward, including ADAPT SERON, were not included. Finally, overlap between patient populations in real‐world studies from the same countries cannot be completely excluded.

## 5 Conclusion

In RCTs, efgartigimod produced significant short‐term improvements in MG‐ADL and QMG scores and approximately doubled the proportion of MG‐ADL responders compared with placebo, with an adverse event incidence comparable to placebo (moderate‐certainty evidence from two manufacturer‐sponsored trials, dominated by the phase 3 ADAPT trial). The benefit was not maintained at the end of follow‐up, supporting cyclical, individualized retreatment. Real‐world studies suggest benefit across serological subtypes, including MuSK‐Ab+ disease, and a corticosteroid‐sparing effect, but this evidence is heterogeneous and of very low certainty. Larger, longer term controlled studies, including trials independent of the manufacturer and particularly in MuSK‐Ab+ and seronegative patients, are needed to confirm the durability of response and to define the optimal retreatment strategy.

## Author Contributions

Conceptualization: **Ghazi Uddin Ahmed** and **Abedin Samadi**. Protocol development and PROSPERO registration: Ghazi Uddin Ahmed and **Jawad Ghias Shaikh**. Literature search: Ghazi Uddin Ahmed and **Sejal Kinger**. Study screening and selection: Sejal Kinger, FNU Ramsha, FNU Prejna, FNU Ritik (two independent reviewers, with disagreements resolved by consensus). Data extraction: **Iqra Shabir**, **Faisal Suhail**, **Muhammad Abdul Basit**, and **Muhammad Moiz** (two independent extractors, with a third reviewer resolving discrepancies). Risk‐of‐bias and quality assessment: **Amjad Ali** and **Ahmed Asad Raza** (two independent reviewers). Formal analysis and GRADE assessment: Ghazi Uddin Ahmed and Jawad Ghias Shaikh. Visualization: Ghazi Uddin Ahmed and FNU Ritik. Writing – original draft: Ghazi Uddin Ahmed, FNU Ramsha, Sejal Kinger, and FNU Prejna. Writing – review and editing: All authors. Supervision and project administration: Abedin Samadi. All authors read and approved the final manuscript and agree to be accountable for all aspects of the work.

## Funding

The authors have nothing to report.

## Ethics Statement

Ethical approval was not required for this study because it is a systematic review and meta‐analysis of previously published studies.

## Conflicts of Interest

The authors declare no conflicts of interest.

## Supporting information




**Supporting Table S1**.: Search strategies for the original searches and for the supplementary searches performed during peer review.
**Supporting Table S2**.: Detailed characteristics of the included studies (study aims, eligibility criteria, and authors’ conclusions).
**Supporting Table S3**.: Leave‐one‐out influence analyses of single‐arm outcomes.
**Supporting Table S4**.: Subgroup analyses by study design and sensitivity analyses restricted to good‐quality cohort studies or excluding the double‐seronegative cohort.
**Supporting Table S5**.: Comparison of the present review with previous systematic reviews and meta‐analyses of efgartigimod in myasthenia gravis.
**Supporting Table S6**.: Study‐level data used in each meta‐analysis.
**Supporting Figure S1**.: Forest plot of mean change in MG‐ADL score in single‐arm studies.
**Supporting Figure S2**.: Forest plot of mean change in QMG score in single‐arm studies.
**Supporting Figure S3**.: Forest plot of the proportion of MG‐ADL responders in single‐arm studies.
**Supporting Figure S4**.: Forest plot of the proportion of QMG responders in single‐arm studies.
**Supporting Figure S5**.: Forest plot of the proportion of responders among AChR‐Ab+ patients in single‐arm studies.
**Supporting Figure S6**.: Forest plot of the proportion of responders among MuSK‐Ab+ patients in single‐arm studies.
**Supporting Figure S7**.: Forest plot of the proportion of patients achieving clinically meaningful improvement in single‐arm studies.
**Supporting Figure S8**.: Forest plot of mean change in prednisolone dose in single‐arm studies.
**Supporting Figure S9**.: Forest plot of the proportion of patients experiencing adverse events in single‐arm studies.

## Data Availability

All data analyzed during this study are included in the published articles referenced in this manuscript and are available from the corresponding author upon reasonable request.
