# Supplementary material for: Safety and Efficacy of Efgartigimod for Generalized Myasthenia Gravis: A Systematic Review and Meta‐Analysis
Source: Brain Behav. 2026 Nov 11;16(11):e71875. doi: 10.1002/brb3.71875 (PMC13753880; doi:10.1002/brb3.71875)
Supplement: Supplementary file 1 [file BRB3-16-e71875-s001.docx]

**Supplementary Material**

Safety and Efficacy of Efgartigimod for Generalized Myasthenia Gravis: A Systematic Review and Meta-Analysis

**Table S1. Search strategies for the original searches (PubMed, Web of Science, and Scopus) and for the supplementary searches performed during peer review (CENTRAL and ClinicalTrials.gov).**

| **Database** | **Search strategy** | **Results** | **Limitations** | **Date of search** |
| --- | --- | --- | --- | --- |
| PubMed | ("Myasthenia Gravis") AND (efgartigimod OR "ARGX-113") (All fields) | 177 | No limitations applied | 8 August 2025 |
| Web of Science | TS= ( "Myasthenia Gravis" ) AND ( efgartigimod OR "ARGX-113" ) | 251 | No limitations applied | 8 August 2025 |
| Scopus | TITLE-ABS-KEY ( "Myasthenia Gravis" ) AND ( efgartigimod OR "ARGX-113" ) | 249 | No limitations applied | 8 August 2025 |
| Cochrane CENTRAL (Cochrane Library)* | #1 MeSH descriptor: [Myasthenia Gravis] explode all trees  #2 ("myasthenia gravis"):ti,ab,kw  #3 #1 OR #2  #4 (efgartigimod OR "ARGX-113" OR "ARGX 113" OR vyvgart):ti,ab,kw  #5 #3 AND #4 (Trials) | 96 | Publication date up to 8 August 2025 | 12 October 2026 |
| ClinicalTrials.gov* | Condition/disease: myasthenia gravis; Intervention/treatment: efgartigimod OR ARGX-113 OR Vyvgart | 21 | Studies first posted up to 8 August 2025 | 12 October 2026 |

*Supplementary searches performed during peer review on 12 October 2026, limited to records up to 8 August 2025, the end date of the original search. The 117 records retrieved were checked against the records identified in the original search and screened against the eligibility criteria; none met the eligibility criteria, and no additional study was included.

**Table S2. Detailed characteristics of the included studies (study aims, eligibility criteria, treatment, endpoints, and authors’ conclusions).**

| Study ID | Country | Study Design | Sample Size | Study Period | Aim of Study | Main Inclusion Criteria | Exclusion Criteria | Treatment & Dosage | Primary Endpoints | Follow-up Duration | Conclusion |
| --- | --- | --- | --- | --- | --- | --- | --- | --- | --- | --- | --- |
| Frangiamore et al. 2024 | Italy | Prospective real-world observational study (EAP) | 19 | Nov 2021 – Jan 2023 | To evaluate the efficacy and safety of efgartigimod in generalized MG patients. | Adults ≥18 years, MGFA class II–IV, MG-ADL ≥5 with >50% nonocular symptoms, COVID-19 negative or vaccinated, IgG ≥6 g/L | Not specified | Efgartigimod 10 mg/kg IV, 4 weekly infusions per cycle for 2 fixed cycles + flexible retreatment | Efficacy of EFG was assessed by the Myasthenia Gravis Activities of Daily Living, Myasthenia Gravis Composite, and Quantitative Myasthenia Gravis scales. | 14 months | EFG was safe and modified significantly the course of the disease along a 14-month follow-up. Our experience strengthens the role of FcRn inhibition as an effective new tool for long-term treatment of gMG. |
| Hao et al. 2025 | China | Single-center real-world retrospective cohort | 77 | Sep 2023 - Jan 2024 | To evaluate the efficacy and safety of efgartigimod in a real-world cohort of patients with AChR+ MG. | AChR+ MG diagnosis, clinical symptoms of oMG or gMG, received efgartigimod | Seropositivity for MuSK or LRP4 antibodies, or triple-seronegative status; abnormal repetitive nerve stimulation findings indicative of other neuromuscular junction disorders; pregnancy. | Efgartigimod 10 mg/kg once a week for 4 weeks per cycle, with subsequent cycles based on clinical evaluation (some received Q2W dosing or multiple cycles). | Mean MG-ADL score changes from baseline at weeks 4, 8, and 12 & prednisone dose and adverse events | 12 weeks | Efgartigimod safely improved MG-ADL scores and reduced steroid use across MG subtypes, with sustained efficacy through multiple treatment cycles. |
| Horiuchi et al. 2024 | Japan | Retrospective case series | 16 | June 2022 – June 2023 | to evaluate symptomatic changes and safety of treatment with efgartigimod in patients with generalized MG (gMG) double-seronegative for acetylcholine receptor antibody and muscle-specific kinase antibody. | Age >18, diagnosis of gMG, negative AChR and MuSK antibodies, efgartigimod treatment | Not specified | Efgartigimod 10 mg/kg IV in cycles. Fixed period (2 cycles) followed by a flexible period. | Symptomatic changes by MG-ADL score change, prednisolone dose, adverse events | ~36.4 weeks | This retrospective study revealed no significant treatment benefit in the outcomes of patients with double-seronegative gMG treated with efgartigimod. |
| Howard et al. 2019 | Belgium, Canada, Italy, Netherlands, Poland, Spain, Sweden, USA | Randomized, double-blind, placebo-controlled, phase 2 | 24 | Dec 2016 - Jul 2017 | To investigate the safety and explore the efficacy of efgartigimod in in AChR+ generalized MG patients. | Adults Age ≥18 with gMG with a history of anti-AChR autoantibodies, MG-ADL ≥5, MGFA Class II–IVa, on stable standard-of-care MG treatment. | History of malignancy, including malignant thymoma, thymectomy <3 months prior to screening, monoclonal antibody for immunomodulation within 3–6 months, IVIg/plasmapheresis within 4 weeks, MGFA Class I/V prior to first dosing. | Efgartigimod 10 mg/kg IV or placebo; 4 doses over a 3-week period. | Safety and tolerability. | 11 weeks | Efgartigimod was safe and well-tolerated. The correlation between reduction of levels of pathogenic IgG autoantibodies and disease improvement suggests that reducing pathogenic autoantibodies with efgartigimod may offer an innovative approach to treat MG. |
| Howard et al. 2021 | North America, Europe, Japan | Randomized, double-blind, placebo-controlled, phase 3 (ADAPT) | 167 | September 5, 2018 and November 26, 2019 | to assess the efficacy and safety of efgartigimod (ARGX-113), a human IgG1 antibody Fc fragment 125 engineered to reduce pathogenic IgG autoantibody levels, in patients with gMG | Adults with gMG, with or without AChR-Ab, MGFA Class II to IV, MG-ADL score ≥5 stable on ≥1 gMG treatment (AChE inhibitors, steroids, NSISTs) | Rituximab or eculizumab in the prior 6 months, thymectomy within 3 months, IVIg or PLEX within 1 month of screening, active hepatitis B, seropositive for hepatitis C, seropositive for HIV with low CD4 count, serum IgG levels <6 g/L at screening, or were pregnant. | Efgartigimod 10 mg/kg IV weekly in cycles of 4 weekly infusions. | Percentage of AChR-Ab+ patients who were MG-ADL responders in C1. | 26 weeks | Efgartigimod was well tolerated and efficacious in patients with gMG. |
| Luo et al. 2024 | China | Multicenter real-world cohort | 61 | Sep–Dec 2023 | Evaluate real-world efficacy/safety of efgartigimod in Chinese gMG patients. | Adult gMG with AChR/MuSK antibodies or RNS positivity; treated at 14 tertiary centers | Not specified | Efgartigimod 10 mg/kg as 4 infusions per cycle. | MG-ADL score reduction, CMI, MSE, immunosuppressants, and the incidence of treatment-emergent adverse events (TEAEs). | 12 weeks | This multicenter cohort study demonstrated the efficacy of efgartigimod in rapid control of gMG. Patients with MGAE or MC would benefit from efgartigimod treatment. |
| Nomura et al. 2024 | Japan | Single-center case series | 16 | Aug 2022 – Sep 2023 | To describe a real-world experience in Japan of efgartigimod treatment for gMG patients with diverse backgrounds. | Age ≥15 years, gMG diagnosis per Japanese guidelines, received ≥1 dose of efgartigimod | Not specified | Efgartigimod 10 mg/kg IV once weekly for 4 weeks in one cycle. | QMG responders (≥ 3 point reduction), IgG levels, and change in prednisolone dose, in the first cycle of efgartigimod. | Up to 13 months | Improvements in the outcomes of patients with gMG, including patients with severe gMG, myasthenic crisis, and refractory to anti-complementary therapy, were observed after the first cycle of efgartigimod treatment. |
| Pane et al. 2024 | Italy | Multicenter retrospective cohort | 63 (31 with efgartigimod) | Sep 2023 – Jan 2024 | Compare real-world efficacy and safety of eculizumab vs efgartigimod in gMG | Adults with gMG initiating eculizumab or efgartigimod treatment in clinical practice | Not specified | Eculizumab: 900 mg IV for 4 weeks + 1200 mg for 2 weeks; Efgartigimod: 10 mg/kg IV weekly for 4 cycle | MG-ADL, QMG, steroid dose, clinical events to both eculizumab and efgartigimod treatments in a real-life setting. | Up to 48 weeks | Eculizumab and efgartigimod can be used in clinical practice to reduce disability in gMG patients. Eculizumab-treated patients had a higher QMG response and steroid sparing effect. Efgartigimod may offer a more flexible schedule. |
| Silvestri et al. 2025 | USA | Retrospective, single-center | 19 | Jan 2022 – Jan 2024 | To describe one center's approach to efgartigimod dosing and its safety and effectiveness in AChR-Ab+ gMG patients. | Adults with AChR-Ab+ gMG with symptoms not adequately controlled by oral medications and/or IVIg. | Not specified | Efgartigimod 10 mg/kg IV in cycles. First 3 cycles at fixed intervals, then individualized. | Effectiveness measured by the Myasthenia Gravis Activities of Daily Living (MG-ADL) scale, MSE, steroid reduction | 6 to 29 months | Individualized dosing of efgartigimod resulted in substantial MG-ADL score improvement, and reduced daily dose and/or discontinuation of concomitant corticosteroids. |
| Suzuki et al. 2024 | Japan | Registry-based observational cohort | 36 | May – Sep 2022 | To assess the efficacy and safety of efgartigimod in patients with gMG. | Patients with generalized MG in the 2021 survey of Japan Myasthenia Gravis Registry (JAMG-R) study group who received an initial cycle of efgartigimod. | Not specified | Efgartigimod 10 mg/kg IV in cycles of 4 weekly infusions as needed avg 2.4 cycles. | MG-ADL improvement, MG composite, MG-QOL15-r. "Responders" defined as patients who achieved a score ≥2 points for MG activities of daily living (MG-ADL) in the first treatment cycle. | 26 weeks | Efgartigimod can be considered for the treatment of patients with generalized MG who do not achieve minimal manifestations, with a broad flexibility of patient selection and treatment schedules. |
| Wang et al. 2024 | China | Prospective cohort study | 14 | Jul 2022 – Aug 2023 | To evaluate the efficacy and safety of efgartigimod across different MG subtypes and treatment cycles in a real-world setting. | Age ≥18, gMG, MGFA II–IV, MG-ADL ≥5, AChR-Ab+, stable treatment for ≥1 month | Rituximab/eculizumab <6 mo, thymectomy <3 mo, IVIg/PE <1 mo, active HBV/HCV/HIV, IgG <6 g/L, pregnancy | Efgartigimod 10 mg/kg IV, once a week for 4 weeks per cycle. | Mean change in MG activities of daily living (MG-ADL) total score from baseline to 4 weeks, QMG, IgG reduction, safety. | 8 weeks | A single cycle of efgartigimod treatment demonstrates effectiveness and the tolerability through week 8, with no new safety signals observed in Chinese gMG patients. |

Studies are listed alphabetically. The eculizumab arm of Pane et al. (n = 32) was not included in any analysis.

**Table S3. Leave-one-out influence analyses of single-arm outcomes. Each row shows the pooled estimate after omitting the named study.**

| **Study omitted** | **Pooled estimate (95% CI)** | **I²** |
| --- | --- | --- |
| **Mean change in MG-ADL score (Figure S1); all studies: -4.97 (-6.44 to -3.50), I² = 86.7%** | | |
| Frangiamore et al. 2024 | -5.07 (-6.79 to -3.36) | 89.0% |
| Hao et al. 2025 | -4.53 (-6.04 to -3.01) | 80.7% |
| Horiuchi et al. 2024 | -5.74 (-6.67 to -4.81) | 60.2% |
| Pane et al. 2024 | -4.95 (-6.76 to -3.14) | 89.1% |
| Silvestri et al. 2025 | -4.72 (-6.45 to -3.00) | 89.0% |
| Wang et al. 2024 | -4.89 (-6.64 to -3.14) | 89.4% |
| **Mean change in QMG score (Figure S2); all studies: -4.83 (-6.69 to -2.97), I² = 55.9%** | | |
| Frangiamore et al. 2024 | -5.37 (-6.48 to -4.26) | 0.0% |
| Pane et al. 2024 | -4.57 (-8.40 to -0.75) | 77.1% |
| Wang et al. 2024 | -4.14 (-6.52 to -1.75) | 64.4% |
| **MG-ADL responders (proportion) (Figure S3); all studies: 0.947 (0.813–0.986), I² = 74.0%** | | |
| Frangiamore et al. 2024 | 0.930 (0.777–0.981) | 78.3% |
| Hao et al. 2025 | 0.927 (0.752–0.981) | 64.9% |
| Luo et al. 2024 | 0.943 (0.752–0.989) | 65.7% |
| Pane et al. 2024 | 0.962 (0.827–0.993) | 78.3% |
| Silvestri et al. 2025 | 0.930 (0.777–0.981) | 78.3% |
| Suzuki et al. 2024 | 0.963 (0.876–0.990) | 51.5% |
| Wang et al. 2024 | 0.959 (0.798–0.993) | 78.1% |
| **QMG responders (proportion) (Figure S4); all studies: 0.744 (0.380–0.932), I² = 65.6%** | | |
| Frangiamore et al. 2024 | 0.816 (0.570–0.937) | 0.0% |
| Nomura et al. 2024 | 0.793 (0.309–0.970) | 75.4% |
| Pane et al. 2024 | 0.780 (0.217–0.978) | 56.2% |
| Wang et al. 2024 | 0.595 (0.364–0.790) | 77.0% |
| **AChR-Ab+ responders (proportion) (Figure S5); all studies: 0.935 (0.744–0.986), I² = 48.5%** | | |
| Frangiamore et al. 2024 | 0.920 (0.708–0.982) | 55.8% |
| Hao et al. 2025 | 0.881 (0.697–0.960) | 55.8% |
| Luo et al. 2024 | 0.932 (0.665–0.990) | 0.0% |
| Nomura et al. 2024 | 0.955 (0.749–0.993) | 55.5% |
| Pane et al. 2024 | 0.955 (0.764–0.993) | 53.5% |
| Silvestri et al. 2025 | 0.910 (0.700–0.978) | 55.8% |
| Suzuki et al. 2024 | 0.956 (0.788–0.992) | 38.8% |
| Wang et al. 2024 | 0.949 (0.707–0.993) | 52.4% |
| **MuSK-Ab+ responders (proportion) (Figure S6); all studies: 0.781 (0.499–0.927), I² = 0.0%** | | |
| Frangiamore et al. 2024 | 0.857 (0.573–0.964) | 0.0% |
| Luo et al. 2024 | 0.714 (0.439–0.889) | 0.0% |
| Nomura et al. 2024 | 0.824 (0.573–0.942) | 0.0% |
| Pane et al. 2024 | 0.733 (0.467–0.896) | 0.0% |
| Suzuki et al. 2024 | 0.798 (0.198–0.985) | 0.0% |
| **Clinically meaningful improvement (proportion) (Figure S7); all studies: 0.884 (0.714–0.959), I² = 81.1%** | | |
| Frangiamore et al. 2024 | 0.913 (0.758–0.973) | 82.7% |
| Hao et al. 2025 | 0.827 (0.653–0.924) | 76.6% |
| Luo et al. 2024 | 0.861 (0.638–0.956) | 77.0% |
| Silvestri et al. 2025 | 0.882 (0.664–0.966) | 84.1% |
| Suzuki et al. 2024 | 0.916 (0.774–0.972) | 76.9% |
| Wang et al. 2024 | 0.888 (0.678–0.968) | 84.7% |
| **Change in prednisolone dose, mg/day (Figure S8); all studies: -10.81 (-16.31 to -5.31), I² = 98.8%** | | |
| Frangiamore et al. 2024 | -10.62 (-17.42 to -3.81) | 99.1% |
| Hao et al. 2025 | -10.00 (-16.81 to -3.19) | 97.3% |
| Horiuchi et al. 2024 | -13.60 (-14.59 to -12.61) | 0.0% |
| Luo et al. 2024 | -10.44 (-17.48 to -3.39) | 99.0% |
| Silvestri et al. 2025 | -9.85 (-15.90 to -3.80) | 99.1% |
| **Adverse events (proportion) (Figure S9); all studies: 0.205 (0.104–0.363), I² = 81.3%** | | |
| Frangiamore et al. 2024 | 0.165 (0.090–0.282) | 73.2% |
| Hao et al. 2025 | 0.250 (0.139–0.407) | 76.2% |
| Horiuchi et al. 2024 | 0.194 (0.090–0.368) | 83.5% |
| Luo et al. 2024 | 0.221 (0.106–0.405) | 80.8% |
| Nomura et al. 2024 | 0.188 (0.088–0.356) | 83.0% |
| Pane et al. 2024 | 0.232 (0.118–0.406) | 81.6% |
| Silvestri et al. 2025 | 0.205 (0.095–0.385) | 83.6% |
| Suzuki et al. 2024 | 0.185 (0.087–0.352) | 81.8% |
| Wang et al. 2024 | 0.214 (0.103–0.393) | 83.3% |

Continuous outcomes are mean changes from baseline (REML random-effects model); proportions were pooled with a generalized linear mixed model (logit link), as in the main analyses.

**Table S4. Subgroup analyses by study design and sensitivity analyses restricted to good-quality cohort studies or excluding the double-seronegative cohort (Horiuchi et al.).**

| **Outcome** | **Cohort studies** | **Case series** | **P for subgroup difference** | **Good-quality cohorts only (NOS ≥ 7)** | **Excluding Horiuchi et al.** |
| --- | --- | --- | --- | --- | --- |
| Mean change in MG-ADL score | -5.59 (-6.79 to -4.39); k = 4 | -3.97 (-8.18 to 0.24); k = 2 | 0.47 | -4.96 (-5.85 to -4.07); k = 3 | -5.74 (-6.67 to -4.81); I² = 60.2% |
| Mean change in QMG score | -4.83 (-6.69 to -2.97); k = 3 | — | NA | -4.83 (-6.69 to -2.97); k = 3 | Not applicable |
| MG-ADL responders (proportion) | 0.930 (0.777–0.981); k = 6 | 1.000 (0.824–1.000); k = 1 (Silvestri) | NA | 0.930 (0.806–0.977); k = 4 | Not applicable |
| QMG responders (proportion) | 0.793 (0.309–0.970); k = 3 | 0.688 (0.413–0.890); k = 1 (Nomura) | NA | 0.793 (0.309–0.970); k = 3 | Not applicable |
| AChR-Ab+ responders (proportion) | 0.933 (0.700–0.988); k = 6 | 0.941 (0.274–0.999); k = 2 | 0.95 | 0.905 (0.717–0.973); k = 4 | Not applicable |
| MuSK-Ab+ responders (proportion) | 0.824 (0.573–0.942); k = 4 | 0.000 (0.000–0.975); k = 1 (Nomura) | NA | 0.895 (0.164–0.997); k = 3 | Not applicable |
| Clinically meaningful improvement (proportion) | 0.882 (0.664–0.966); k = 5 | 0.895 (0.669–0.987); k = 1 (Silvestri) | NA | 0.860 (0.638–0.955); k = 3 | Not applicable |
| Change in prednisolone dose, mg/day | -13.53 (-14.61 to -12.44); k = 3 | -8.35 (-23.57 to 6.87); k = 2 | 0.51 | -12.56 (-14.52 to -10.60); k = 2 | -13.60 (-14.59 to -12.61); I² = 0.0% |
| Adverse events (proportion) | 0.169 (0.061–0.389); k = 6 | 0.294 (0.186–0.432); k = 3 | 0.28 | 0.188 (0.054–0.483); k = 4 | 0.194 (0.090–0.368); I² = 83.5% |

k, number of studies; NA, not applicable (test not performed when a subgroup contained fewer than two studies). For single-study subgroups, the study proportion is shown with its exact (Clopper-Pearson) 95% CI.

**Table S5. Comparison of the present review with previous systematic reviews and meta-analyses of efgartigimod in myasthenia gravis.**

| **Review** | **Sources searched (end date)** | **Designs and size** | **Efgartigimod evidence and outcomes** | **Randomized vs observational evidence** | **MuSK-Ab+ pooled** | **Steroid dose pooled** | **GRADE** |
| --- | --- | --- | --- | --- | --- | --- | --- |
| Saccà et al. 2023 (ref. 17) | PubMed, Cochrane Library, AAN/EAN/MGFA conference abstracts (to 31 December 2022) | Double-blind placebo-controlled RCTs; 7 RCTs of 6 drugs (pairwise and network meta-analysis) | ADAPT only; MG-ADL, QMG, MGC, and MG-QoL15 at each trial's primary time point | RCTs only | No | No | No |
| Zhong et al. 2024 (ref. 19) | PubMed, Web of Science, CENTRAL, ClinicalTrials.gov (to 20 January 2024) | Phase II/III placebo-controlled RCTs; 12 RCTs of 9 biological therapies (network meta-analysis) | ADAPT only; QMG change at 1 and 4 weeks, at maximal response, and 4 weeks after the last dose | RCTs only | No | No | No |
| Li et al. 2024 (ref. 18) | PubMed, Embase, Cochrane Library, ClinicalTrials.gov (to 18 May 2023) | RCTs of 4 FcRn inhibitors; 6 RCTs, 532 participants | Efgartigimod-specific estimate reported for MG-ADL responders only | RCTs only | No | No | No |
| Cheng et al. 2025 (ref. 20) | PubMed, Embase, Web of Science, Cochrane Library, ClinicalTrials.gov (to 25 September 2024; English only) | RCTs, cohort studies, and single-arm trials; 10 studies, 305 patients (refractory MG) | 2 RCTs and 8 observational studies; pooled response rate (MG-ADL reduction of ≥2 points at 4 weeks) and adverse events | Pooled across designs, with subgroup analysis by design and a separate analysis of RCTs | No (AChR-Ab+ vs non-differentiated only) | No | No |
| Present review | PubMed, Scopus, Web of Science (to 8 August 2025), supplemented by CENTRAL and ClinicalTrials.gov (searched 12 October 2026; same end date) | 2 RCTs, 6 cohort studies, 3 case series; 11 studies, 480 participants | 2 RCTs and 9 observational studies; MG-ADL and QMG at peak effect and at end of follow-up, responders, AChR-Ab+ and MuSK-Ab+ responders, corticosteroid dose, adverse events; prediction intervals | Separate tiers: two-arm analyses of RCTs only; single-arm analyses of observational studies | Yes (5 studies, 18 patients) | Yes (5 studies) | Yes |

AAN, American Academy of Neurology; CENTRAL, Cochrane Central Register of Controlled Trials; EAN, European Academy of Neurology; MGC, Myasthenia Gravis Composite; MGFA, Myasthenia Gravis Foundation of America; MG-QoL15, 15-item Myasthenia Gravis Quality of Life scale; RCT, randomized controlled trial. 'No' indicates that no such analysis was reported. Information was extracted from the published report of each review; reference numbers refer to the main manuscript. Five of the nine observational studies in the present review (Pane et al. 2024, Hao et al. 2025, Suzuki et al. 2024, Silvestri et al. 2025, and Nomura et al. 2024) were not included in Cheng et al., the only review in this table that pooled observational data.

**Table S6. Study-level data used in each meta-analysis.**

| **Outcome** | **Study** | **Efgartigimod** | **Placebo** | **Weight (%)** |
| --- | --- | --- | --- | --- |
| **A. Randomized controlled trials: two-arm analyses (Figures 3–5); continuous outcomes are mean change from baseline (SD); n** | | | | |
| MG-ADL change, peak effect | Howard et al. 2019 | -4.4 (3.46); 12 | -2.9 (4.16); 12 | 20.0 |
|  | Howard et al. 2021 (ADAPT) | -4.9 (4.84); 65 | -2.0 (4.00); 64 | 80.0 |
| MG-ADL change, end of follow-up | Howard et al. 2019 | -3.5 (4.16); 12 | -1.5 (4.50); 12 | 21.4 |
|  | Howard et al. 2021 (ADAPT) | -1.6 (4.84); 65 | -1.0 (5.60); 64 | 78.6 |
| QMG change, peak effect | Howard et al. 2019 | -5.7 (4.16); 12 | -2.1 (3.46); 12 | 29.1 |
|  | Howard et al. 2021 (ADAPT) | -6.2 (6.45); 65 | -1.2 (4.80); 64 | 70.9 |
| QMG change, end of follow-up | Howard et al. 2019 | -4.8 (5.20); 12 | -1.5 (4.16); 12 | 18.7 |
|  | Howard et al. 2021 (ADAPT) | -2.0 (5.64); 65 | -0.8 (4.80); 64 | 81.3 |
| MG-ADL responders (and CMI) | Howard et al. 2019 | 9/12 | 3/12 | 8.5 |
|  | Howard et al. 2021 (ADAPT) | 57/84 | 31/83 | 91.5 |
| AChR-Ab+ responders | Howard et al. 2019 | 9/12 | 3/12 | 13.8 |
|  | Howard et al. 2021 (ADAPT) | 44/65 | 19/64 | 86.2 |
| Adverse events | Howard et al. 2019 | 10/12 | 10/12 | 14.6 |
|  | Howard et al. 2021 (ADAPT) | 65/84 | 70/83 | 85.4 |
| **B. Observational studies: single-arm analyses (Figures S1–S9)** | | | | |
| MG-ADL change, mean change (SE); n | Frangiamore et al. 2024 | -4.30 (1.1241); 19 | – | – |
|  | Hao et al. 2025 | -6.90 (0.4336); 76 | – | – |
|  | Horiuchi et al. 2024 | -1.80 (0.7500); 16 | – | – |
|  | Pane et al. 2024 | -5.00 (0.5747); 31 | – | – |
|  | Silvestri et al. 2025 | -6.10 (0.6306); 17 | – | – |
|  | Wang et al. 2024 | -5.36 (0.9996); 14 | – | – |
|  | Pooled (random effects) | -4.97 (-6.44 to -3.50); 95% PI -10.05 to 0.11; tau² = 2.79; I² = 86.7% | – | – |
| QMG change, mean change (SD); n | Frangiamore et al. 2024 | -2.60 (5.90); 19 | – | – |
|  | Pane et al. 2024 | -5.10 (3.50); 31 | – | – |
|  | Wang et al. 2024 | -6.50 (4.80); 14 | – | – |
|  | Pooled (random effects) | -4.83 (-6.69 to -2.97); 95% PI -24.83 to 15.17; tau² = 1.58; I² = 55.9% | – | – |
| MG-ADL responders, n/N | Frangiamore et al. 2024 | 19/19 | – | – |
|  | Hao et al. 2025 | 75/76 | – | – |
|  | Luo et al. 2024 | 59/61 | – | – |
|  | Pane et al. 2024 | 25/31 | – | – |
|  | Silvestri et al. 2025 | 19/19 | – | – |
|  | Suzuki et al. 2024 | 21/34 | – | – |
|  | Wang et al. 2024 | 12/14 | – | – |
|  | Pooled (random effects) | 0.947 (0.813-0.986); 95% PI 0.190-0.999; tau² = 2.33; I² = 74.0% | – | – |
| QMG responders, n/N | Frangiamore et al. 2024 | 6/19 | – | – |
|  | Nomura et al. 2024 | 11/16 | – | – |
|  | Pane et al. 2024 | 23/31 | – | – |
|  | Wang et al. 2024 | 14/14 | – | – |
|  | Pooled (random effects) | 0.744 (0.380-0.932); 95% PI 0.003-1.000; tau² = 1.90; I² = 65.6% | – | – |
| AChR-Ab+ responders, n/N | Frangiamore et al. 2024 | 8/8 | – | – |
|  | Hao et al. 2025 | 76/76 | – | – |
|  | Luo et al. 2024 | 54/56 | – | – |
|  | Nomura et al. 2024 | 10/14 | – | – |
|  | Pane et al. 2024 | 15/22 | – | – |
|  | Silvestri et al. 2025 | 19/19 | – | – |
|  | Suzuki et al. 2024 | 11/19 | – | – |
|  | Wang et al. 2024 | 12/14 | – | – |
|  | Pooled (random effects) | 0.935 (0.744-0.986); 95% PI 0.091-1.000; tau² = 3.47; I² = 48.5% | – | – |
| MuSK-Ab+ responders, n/N | Frangiamore et al. 2024 | 2/4 | – | – |
|  | Luo et al. 2024 | 4/4 | – | – |
|  | Nomura et al. 2024 | 0/1 | – | – |
|  | Pane et al. 2024 | 3/3 | – | – |
|  | Suzuki et al. 2024 | 5/6 | – | – |
|  | Pooled (random effects) | 0.781 (0.499-0.927); 95% PI 0.257-0.974; tau² = 0.12; I² = 0.0% | – | – |
| Clinically meaningful improvement, n/N | Frangiamore et al. 2024 | 12/19 | – | – |
|  | Hao et al. 2025 | 75/76 | – | – |
|  | Luo et al. 2024 | 58/61 | – | – |
|  | Silvestri et al. 2025 | 17/19 | – | – |
|  | Suzuki et al. 2024 | 21/34 | – | – |
|  | Wang et al. 2024 | 12/14 | – | – |
|  | Pooled (random effects) | 0.884 (0.714-0.959); 95% PI 0.148-0.997; tau² = 1.53; I² = 81.1% | – | – |
| Prednisolone dose, mean change (SE), mg/day | Frangiamore et al. 2024 | -12.20 (3.2533) | – | – |
|  | Hao et al. 2025 | -13.90 (0.5506) | – | – |
|  | Horiuchi et al. 2024 | -1.30 (0.4700) | – | – |
|  | Luo et al. 2024 | -12.60 (1.0499) | – | – |
|  | Silvestri et al. 2025 | -16.90 (4.8636) | – | – |
|  | Pooled (random effects) | -10.81 (-16.31 to -5.31); 95% PI -31.37 to 9.75; tau² = 33.88; I² = 98.8% | – | – |
| Adverse events, n/N | Frangiamore et al. 2024 | 13/19 | – | – |
|  | Hao et al. 2025 | 3/76 | – | – |
|  | Horiuchi et al. 2024 | 5/16 | – | – |
|  | Luo et al. 2024 | 7/61 | – | – |
|  | Nomura et al. 2024 | 6/16 | – | – |
|  | Pane et al. 2024 | 2/31 | – | – |
|  | Silvestri et al. 2025 | 4/19 | – | – |
|  | Suzuki et al. 2024 | 13/34 | – | – |
|  | Wang et al. 2024 | 2/14 | – | – |
|  | Pooled (random effects) | 0.205 (0.104-0.363); 95% PI 0.016-0.799; tau² = 1.18; I² = 81.3% | – | – |

Data are those entered in the analyses shown in Figures 3–5 (main manuscript) and Figures S1–S9. Weights refer to the random-effects two-arm analyses. Single-arm proportions were pooled with a generalized linear mixed model (logit link) and continuous outcomes with restricted maximum-likelihood random-effects models; pooled estimates are shown with 95% CIs, 95% prediction intervals (PI; t distribution with k − 2 degrees of freedom), tau², and I². CMI, clinically meaningful improvement; SD, standard deviation; SE, standard error.

**Figure S1. Forest plot of mean change in MG-ADL score in single-arm studies.**

**Figure S2. Forest plot of mean change in QMG score in single-arm studies.**

**Figure S3. Forest plot of the proportion of MG-ADL responders in single-arm studies.**

**Figure S4. Forest plot of the proportion of QMG responders in single-arm studies.**

**Figure S5. Forest plot of the proportion of responders among AChR-Ab+ patients in single-arm studies.**

**Figure S6. Forest plot of the proportion of responders among MuSK-Ab+ patients in single-arm studies.**

**Figure S7. Forest plot of the proportion of patients achieving clinically meaningful improvement in single-arm studies.**

**Figure S8. Forest plot of mean change in prednisolone dose (mg/day) in single-arm studies.**

**Figure S9. Forest plot of the proportion of patients experiencing adverse events in single-arm studies.**
